# Structural diversity in the atomic resolution 3D fingerprint of the titin M-band segment

**DOI:** 10.1371/journal.pone.0226693

**Published:** 2019-12-19

**Authors:** Spyros D. Chatziefthimiou, Philipp Hornburg, Florian Sauer, Simone Mueller, Deniz Ugurlar, Emma-Ruoqi Xu, Matthias Wilmanns

**Affiliations:** 1 European Molecular Biology Laboratory, Hamburg Unit, Hamburg, Germany; 2 University Hamburg Medical Centre Hamburg-Eppendorf, Hamburg, Germany; University of Queensland, AUSTRALIA

## Abstract

In striated muscles, molecular filaments are largely composed of long protein chains with extensive arrays of identically folded domains, referred to as “beads-on-a-string”. It remains a largely unresolved question how these domains have developed a unique molecular profile such that each carries out a distinct function without false-positive readout. This study focuses on the M-band segment of the sarcomeric protein titin, which comprises ten identically folded immunoglobulin domains. Comparative analysis of high-resolution structures of six of these domains ‒ M1, M3, M4, M5, M7, and M10 ‒ reveals considerable structural diversity within three distinct loops and a non-conserved pattern of exposed cysteines. Our data allow to structurally interpreting distinct pathological readouts that result from titinopathy-associated variants. Our findings support general principles that could be used to identify individual structural/functional profiles of hundreds of identically folded protein domains within the sarcomere and other densely crowded cellular environments.

## Introduction

Sarcomeric filaments in skeletal and cardiac muscles are one of the most complex and ordered cellular structures, which enable them to perform their extraordinary function of alternating contraction and relaxation. Various proteinaceous filaments that constitute the sarcomeric ultrastructure are formed either by repetitive arrays of identical subunits, as found in actin and myosin filaments, or by very long polypeptide chains that are composed of extensive arrays of domains with an identical fold [1,2]. However, these domains often have unique functional fingerprints and the underlying structural basis of these different readouts is yet to be determined. Structural predictions often only provide limited and insufficiently validated information about details of specific structural features at the atomic level.

Titin has a molecular weight of more than 3 MDa in its longest isoform and is over 1.5 μm in length, spanning half of a sarcomeric unit from the peripheral Z-disk to the central M-band[3–5]. Its almost 300 folded domains are mostly limited to immunoglobulin (Ig) domains or fibronectin type 3 domains, posing a major challenge–one may say “titanic”–to assign specific functions to individual domains. The far C-terminal segment of titin is of particular functional relevance, as it contains the only known catalytic domain with protein kinase activity under *in vitro* conditions [6–8]. This segment is involved in regulating specific downstream signaling pathways and serves as a hub for various interactions with other sarcomeric filaments within the central M-band [3,7,9,10].

**Fig 1:**
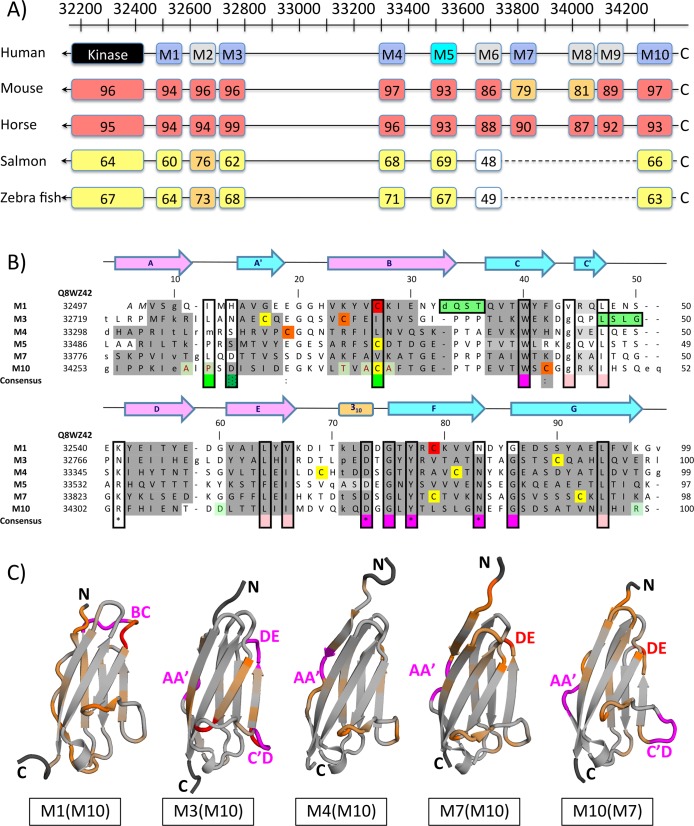
Sequence and structural diversity in titin M-band Ig domains. A) M-band domain organization in representative titin sequences from Homo sapiens (Q8WZ42), mouse (A2ASS6), horse (F6VG02), Atlantic salmon (A0A1S3PNI2), and zebrafish (A5X6X5). The preceding titin kinase domain has been included as a point of reference. The residue numbers indicated above correlate to the human titin sequence. Those domains for which high-resolution structures have been determined are colored in blue (X-ray) and cyan (NMR). M-band domains M7, M8, and M9 are missing in the sequences from Atlantic salmon and zebrafish. The length of the specified M-band domains and connecting loop regions are proportional to the human titin sequence. Calculated overall sequence identities, using the human titin sequence as reference, are indicated in % and are highlighted by a color spectrum: > 85%, red; >70%, orange; >50%, yellow; <50%, no color. B) Structure-based alignment of the M1, M3, M4, M5, M7, and M10 human titin sequences. Aligned residues are in upper case; residues with an uncertain alignment are in lower case. The positions of the secondary structural elements taken from the M1 structure are shown on top of the alignment. Secondary structural elements for the different M-band sequences, as extracted from the respective structures, are highlighted in grey in the respective sequences. M1 domain residues are numbered according to the established renumbering scheme **(S3 Table)** and are indicated on top of the alignment. Notice that numbers of aligned residues from other domains differ slightly due to gaps in the sequence. For cross checking purposes, residue numbers referring to the complete human titin sequence Q8WZ42 (UniProt) are also shown to the left. Cysteine positions are colored as in **Fig 2**. Residue positions that are invariant or conserved across different M-band domain sequences are boxed and highlighted in the “consensus” line in magenta and salmon, respectively, whereas residue positions that are distinct across different human titin M-band domain sequences but highly conserved for the same M-band domain across different species are in green. Sequence motifs of individual M-band domains that structurally deviate from other M-band domain structures (*cf*. panel C) are also highlighted in green. C) Cartoon representations of the structure of M-band domains M1, M3, M4, M7, and M10, highlighting structural diversity, estimated from pairwise spatial distance plots (**S4 Fig).** The color spectrum ranges from grey to orange to red, reflecting pairwise spatial distances between 1Å and 4Å. Termini outside of superimposable parts of the structures are in dark grey. Sequence segments representing domain-specific sequence insertions are shown in magenta. Identified structural diversity hotspots (red, magenta) are labeled in all M-band domains shown.

Taking titin kinase as the N-terminal boundary of the titin M-band segment, there are another ten Ig domains towards the far C-terminus of titin, referred to as M1 to M10, encoded by at least six exons 358–363, or Mex1 to Mex6, on the respective *TTN* gene [11] **(Fig 1A)**. In contrast to other titin sequence segments that show an overarching pattern of regular domain repetition and clustering [12], the M-band Ig domains are positioned at irregular sequence intervals. Since they are separated by linkers of variable lengths, we do not expect an extensive pattern of direct Ig domain/domain interactions, as for instance observed in the titin segment preceding the kinase domain [13,14]. Some of these linkers are associated with specific binding functions and are subject for posttranslational modification sites (for further details see below). These irregular spacings suggest that any specific functional readout is determined at the level of single titin M-band domains. The degree of sequence conservation for equivalent M-band titin domains is generally > 90% for higher vertebrates and >50% for more distantly related organisms such as zebrafish and Atlantic salmon **(Fig 1A)**. The findings presented in this contribution are therefore generally applicable to all titin sequences with conserved M-band domain structure.

To date, very little is known about the specific function and underlying structures of M1 to M10, except for an NMR structure of the M5 domain and crystal structures of the M10 domain bound to two closely related binding partners, obscurin and obscurin-like 1 (Obsl1). This interaction connects the titin C-terminus to other M-band filament systems such as myomesin and myosin via a nearby obscurin/myomesin binding site [15–20]. The M10 domain has also been reported to interact with myospryn, a muscle-specific protein of the tripartite motif superfamily that mediates interactions with the protease calpain 3 as well as synemin, which functions as an A-kinase anchoring protein [21,22]. Calpain 3 has also been shown to directly interact with the same titin segment as myospryn, including a sequence region covering M-band domains 9 and 10 [23]. The latter interactions with calpain 3, myospryn, and synemin seem to link the titin C-terminus to specific signaling pathways. How exactly these protein ligands bind to the M10 domain and nearby regions, and whether the interactions are overlapping or complementary with the obscurin / Obsl1-binding site, have not yet been determined. Another interaction between the long linker connecting domains M3 and M4 and DRAL/FHL-2, a member of the LIM domain protein subfamily, has also been reported [24] but not yet characterized in mechanistic detail. In addition, the linker between M-band domains 5 and 6 contains an array of KSP motifs that have been shown to be targets for multiple phosphorylation in developing muscle cells [25]. Upon phosphorylation these motifs generate binding sites to a negative regulator of c-Myc activation Bin1 [26]. How all these molecular interactions are embedded into the hexagonal transverse M-band lattice driven mainly by myosin (thick) filaments, remains largely elusive [27].

In parallel, next generation sequencing data from patients with congenital myopathy ‒ the most common form of inherited non-dystrophic muscle disorder in childhood ‒ have revealed relatively frequent mutations in the M-band segment of *TTN* [28]. A substantial number of these *TTN* variants are either homozygous or compound heterozygous and lead either to truncated M-band titin filaments or, more rarely, to titin variants with M-band segment single-residue mutations. The phenotypic interpretation of these data has identified four new titinopathies, in addition to previously identified hereditary myopathies in skeletal muscles and isolated dilated or hypertrophic cardiomyopathy (DCM, HCM) [28,29]. Related disease phenotypes are also found in variants of the M-band binding ligands calpain 3 and myospryn, pointing to a crucial role of calpain 3-mediated degradation and potentially muscle remodeling [22,30]. Knowledge about the underlying functional and structural diversity of the M-band segment of titin is therefore essential for the mechanistic understanding of the corresponding genotype/phenotype relationships.

Expanding on previous structures of the M-band framework domains titin kinase and M10 in complex with obscurin / Obsl1 [6,15,16,20], we have determined the high-resolution structures of five of the titin M-band Ig domains. Analysis of these structures has allowed the identification and characterization of localized diversity hotspots in the two tip regions of these domains. Our data reveal a considerable level of structural diversity for a limited set of identically folded domains within the C-terminal M-band segment of titin. We used these structures to map recently identified titinopathy and DCM/HCM variants that are suggestive to what extent disease-causing effects can be rationalized based on structural findings.

## Materials and methods

### Protein expression and purification

The DNA sequences encoding the studied titin domains were amplified by PCR from complementary DNA (titin, DKFZp451a172q). For information about the residue boundaries of each construct, the vectors and the *Escherichia coli* strains used for the expression, see **S1 Table**. Cells were grown at 37°C in lysogeny broth (LB-Lennox) or terrific broth (TB) medium supplemented with the appropriate antibiotics. Overexpression was induced by addition of 0.5–1 mM isopropyl-β-D-thiogalactopyranoside at an OD_600_ of 0.6 and 3.0 for LB and TB, respectively, and cells were harvested after continuous shaking at 20°C overnight. For purification, the cells were resuspended in lysis buffer, containing 25 mM Tris/HCl (pH 8.0), 200–300 mM NaCl, 5–20 mM imidazole and 1mM tris(2-carboxyethyl)phosphine, sonicated and centrifuged at 18,000 rpm for 30‒60 min at 4°C. The supernatant was filtered and loaded onto nickel-nitrilotriacetic acid (Ni-NTA) beads, which had been equilibrated in lysis buffer. After the Ni-NTA affinity chromatography step, the hexa-histidine tag was cleaved using the appropriate protease. Each protein was further purified by size exclusion chromatography, using a Superdex 75 16/60 column (GE Healthcare). The buffers used for this last purification step were 25 mM Bis-Tris-Propane (pH 7.4) and 50 mM NaCl for M1, 25 mM Tris (pH 8.0), 200 mM NaCl and 2 mM β-mercaptoethanol for M3, and 10 mM Tris (pH 8.0), 150 mM NaCl and 1 mM TCEP for M4, M7 and M10.

### DTNB assay

For the quantification of sulfhydryl groups, a commercially available DTNB-Thiols Assay Kit (Abnova) was used according to the manufacturer’s protocol. M3, M4, M7, and M10 domains were purified under non-reducing conditions and concentrated to 20‒50 μM. Measurements were performed in triplicates using a Tecan Infinite M1000 (Tecan) microplate reader.

### X-ray structure determination of M-band titin domains

Crystals of titin M-band Ig domains were grown by vapor diffusion by mixing equal volumes of protein and precipitant solutions. For M1, crystals grew in 2.2 M ammonium sulfate and 0.1 M Bis-Tris (pH 7.0) at a protein concentration of 8 mg/mL. M3 was crystallized at a protein concentration of 8 mg/mL in 0.2 M ammonium sulfate and 23% [w/v] polyethylene glycol 4000. M4 crystallized in 0.2M Na-citrate (pH 4.0), 1.2M ammonium sulfate and 0.3M magnesium sulfate at a protein concentration of 10mg/mL (tetragonal crystal form) and in 0.1 M MES (pH 6.5), 2.0 M RbBr at a protein concentration of 20mg/mL (trigonal crystal form). For M7, the precipitant solution was 0.1M Tris (pH 8.0), 3.2M ammonium sulfate and 1mM TCEP at a protein concentration of 8 mg/mL. M10 was crystallized in 0.1M Bis Tris (pH 6.8), 55% [v/v] MPD, 0.2M magnesium sulfate at a protein concentration of 40 mg/mL.

X-ray datasets were collected at X11 and X12 beamlines at EMBL/DESY, Hamburg (DORIS storage ring) for M1 and M7, respectively, and at the European Synchrotron Radiation Facility beamlines ID23-1 and ID14-1, Grenoble, France, at 100K, for M3 and M4/M10, respectively. Data were processed with XDS [31] and scaled using XSCALE (M1, M7, M10), SCALA [32] **(**M4) and Aimless [33] (M3).

The structure of the titin M1 domain was solved by molecular replacement using CaspR [34] with a model generated by MODELLER [35], based on the immunoglobulin domains of telokin (1fhg), titin M5 (1tnm) and titin I1 (1g1c). It was refined with REFMAC5 [36] at a resolution of 1.69 Å. There is one molecule in the asymmetric unit and the final model consists of the complete M1 domain plus two additional residues from the cleaved tag at its N-terminus and 129 water molecules.

The structure of the titin domain M3 was solved by molecular replacement with Phaser [37] using telokin (1fhg) as the search model. Model building was done using Autobuild [38], and the structure was refined using the phenix.refine suite [39]. The structure contains four titin M3 molecules per asymmetric unit, forming a tetrameric arrangement of two dimers of dimers. Each of the two M3 domain dimers is formed by an intermolecular β-sheet symmetrically meditated by β-strand D of the two participating M3 domain molecules **(S1 Fig).** The final model also includes eight sulfate ions and 188 water molecules.

The structure of the M4 domain was solved in two different crystal forms. The first one crystallized in a trigonal space group and experimental phases were determined with the single anomalous dispersion (SAD) method, using the anomalous signal of bromide ions from the crystallization buffer. The heavy atom structure was determined with SHELXD [40] and phases were calculated with SHELXE [41], which allowed building a nearly complete model (96/103 residues) with ARP/wARP [42]. The model was completed manually using COOT and refined with phenix.refine [39] to an R_work_ /R_free_ = 0.26/0.28. To facilitate further model refinement, RbBr was exchanged to 2.0 M NaCl by soaking the crystals in a solution containing 0.1 M MES pH 6.5, 2M NaCl supplemented with 20% [v/v] glycerol, immediately before flash freezing. The final M4 model was obtained from a subsequent X-ray data set to 1.60 Å resolution. The absence of RbBr was confirmed by the lack of a measurable X-ray fluorescence signal at the corresponding elemental absorption edges. Interestingly, a broader X-ray fluorescence scan revealed the presence of zinc in the crystal.

The M4 domain structure in the tetragonal crystal form was solved by molecular replacement using Phaser [37] and the trigonal form of M4 as the search model. The structure was refined with phenix.refine [39] at a resolution of 2.0 Å. It contains three M4 domain copies, including an N-terminal glycine residue originating from a 3C-cleavage site per asymmetric unit **(S1 Fig)**, plus 210 water molecules and seven sulfate ions.

The structure of M7 was solved by molecular replacement with Phaser [37], using the structure of telokin (1FHG) as the search model. The structure was refined by phenix.refine [39] at a resolution of 0.96 Å. The asymmetric unit contains one M7 molecule covering the complete domain fragment of M7 except for the very C-terminal residue and one additional residue from the cleaved tag at its N-terminus, two sulfate ions and 160 water molecules.

The structure of M10 was solved by molecular replacement with Phaser [37], using the M10 domain coordinates from the obscurin–titin M10 domain complex (3knb) as the search model. It was refined with phenix.refine [39] at a resolution of 2.0 Å. The asymmetric unit contains two M10 molecules **(S1 Fig),** with the final model composed of the complete domain fragment of M10, four sulfate ions and 152 water molecules.

Detailed X-ray data collection and refinement statistics are summarized in **Table 1.** Ramachandran statistics are calculated using MolProbity [43]

**Table 1:** X-ray structure statistics.

M-band domain	M1	M3	M4 (1)	M4 (2)	M7	M10
**PDB ID**	2bk8	6hci	3qp3	6h4l	3puc	3q5o
**Year of Deposition**	2005	2019	2011	2019	2010	2010
**Data collection**						
**Resolution range** ^a^	17.74–1.69 (1.80–1.69)	30.62–2.12 (2.20–2.12)	48.60–2.00 (2.07–2.00)	27.77–1.60 (1.69–1.60)	19.52–0.96 (0.99–0.96)	39.37–2.05 (2.12–2.05)
**Space group**	I4_1_	P 3_2_21	P4_1_2_1_2	P 3_2_21	P12_1_1	P6_3_22
**Unit cell [Å,°]**	a,b = 68.8 c = 48.2	a,b = 106.1 c = 75.5	a,b = 63.5 c = 151.0	a,b = 84.9 c = 49.2	a = 22.8 b = 68.5 c = 24.2 β = 100.9	a,b = 118.8 c = 85.2
**Total reflections** ^a^	94407 (16296)	133552 (8073)	205861 (19275)	307132 (20252)	88104 (8801)	262847 (21729)
**Unique reflections** ^a^	12629 (2168)	27434 (2320)	21684 (2102)	27114 (3942)	44330 (4432)	22724 (2188)
**Multiplicity** ^a^	7.5 (7.5)	4.9 (3.5)	9.5 (9.1)	11.3 (5.1)	2.0 (2.0)	11.6 (9.8)
**Completeness** ^a^**(%)**	99.6 (99.9)	97.5 (83.7)	99.6 (99.6)	99.6 (99.9)	99.6 (99.8)	99.3 (97.9)
**Mean I/σ(I)** ^a^	27.8 (5.3)	10.0 (1.3)	22.5 (4.6)	24.9 (3.1)	14.2 (4.3)	23.3 (5.6)
**R-merge**^a^	0.042 (0.438)	0.120 (0.888)	0.098 (0.629)	0.051 (0.456)	0.026 (0.177)	0.066 (0.402)
**R-meas**^a^	0.045 (0.470)	0.134 (1.022)	0.104 (0.666)	0.053 (0.505)	0.0362 (0.250)	0.0687 (0.425)
**R-pim** ^a^	0.021 (0.204)	0.0597 (0.490)	0.0330 (0.214)	0.015 (0.203)	0.026 (0.177)	0.0201 (0.135)
**CC1/2** ^a^	0.999 (0.953)	0.996 (0.529)	0.999 (0.903)	1.000 (0.941)	0.998 (0.900)	0.999 (0.976)
**Refinement**						
**R-work** ^a^	0.189 (0.327)	0.198 (0.285)	0.177 (0.194)	0.208 (0.247)	0.116 (0.166)	0.189 (0.277)
**R-free** ^a^^,^ ^b^	0.234 (0.366)	0.241 (0.306)	0.213 (0.217)	0.221 (0.249)	0.135 (0.185)	0.215 (0.287)
**Number of atoms**	918	3356	2636	968	969	1663
**Macromolecules**	789	3128	2310	769	799	1491
**Ligands**		40	35	4	10	20
**Solvent**	129	188	291	195	160	152
**RMSD bonds [Å]**	0.015	0.005	0.007	0.006	0.012	0.007
**RMSD angles [°]**	1.41	1.07	1.03	1.13	1.43	1.03
**Ram. favored [%]** ^**c**^	96.8	98.7	100	100	99	99
**Ram. allowed [%]** ^**c**^	3.2	1.3	0	0	1	1
**Aver** **. B-factor [Å**^**2**^**]**	37.4	39.4	24.2	35.5	12.0	42.1

^a^ Values in parentheses are for the highest resolution shell

^b^ R-free was calculated on a random test set comprising 5% of the data excluded from refinement

RMSD, root-mean-square deviation

^c^ Ramachandran statistics calculated using MolProbity.

### Structural models of M-band domains M2, M6, M8, and M9

Suitable structural templates for homology models were initially searched by using the template search engine, provided by SWISS-MODEL protein structure homology-modeling server [44]. For all four domains, crystal structures of titin M-band domains were among the top homologous sequences. Therefore, we exclusively used those domains as templates for the homology models of M2, M6, M8, and M9. Structural models were subsequently built using the SWISS-MODEL protein structure homology-modeling server. Estimated Sequence similarity for all templates used was > 30%, coverage > 90%, and the Global Model Quality Estimate (GMQE), according to the SWISS-MODEL server was > 0.6. Statistics on the models are summarized in **S2 Table**.

### Estimation of cross-species sequence conservation of M-band domains

The level of sequence conservation of titin M-band domains was estimated by comparing the sequences of human titin (Q8WZ42) with representative sequences from mouse (A2ASS6), horse (F6VG02), Atlantic salmon (A0A1S3PNI2), and zebrafish (A5X6X5) by generating a multiple sequence alignment using Clustal (version 1.2.4) [45]. As a selection criterion for sequences to be compared with the human titin sequence, the overall length of the titin sequences as found in UniProt [46] was used. Only sequences with a similar overall length were chosen, in case of multiple genes (e.g. zebrafish) the one with a length closest to the human sequences was used. To allow correct assignment of M-band domain sequences to specific M-band domains following the established topology of human titin (Q8WZ42) as annotated in UniProt, especially for sequences with missing M-band domains, a sequence identity matrix was calculated with Clustal2.1, and individual M-band domain sequences were assigned to the M-band domain of human titin with the highest identity.

## Results

### High-resolution structures of individual M-band domains

**Fig 2:**
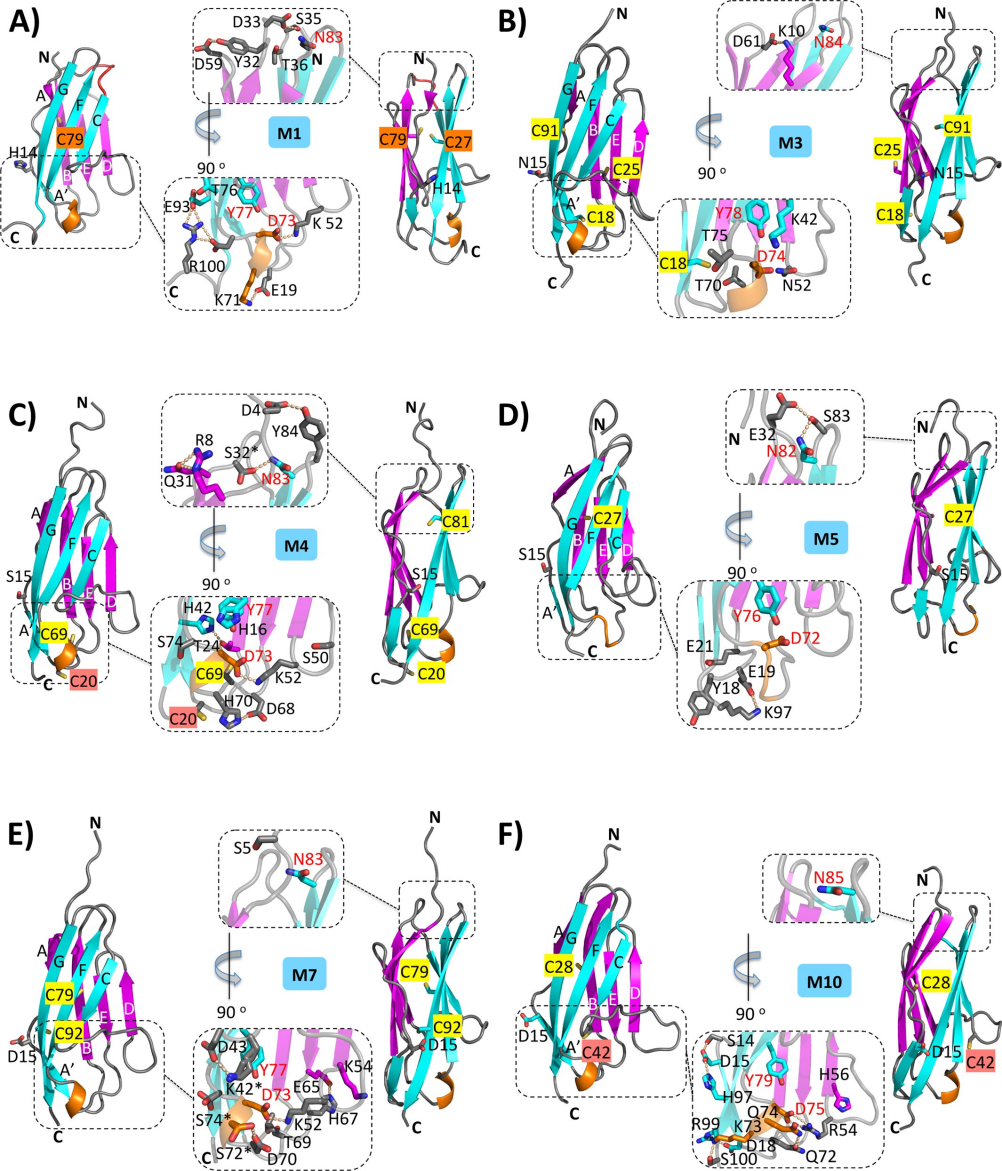
**High-resolution structures of M-band domains A) M1, B) M3, C) M4, D) M5, E) M7 and F) M10.** Cartoon representations in two different orientations; the second view is rotated by approximately 90° around a vertical axis. The two opposite β-sheets ABDE and A’CC’FG are colored in magenta and cyan, respectively. The conserved 3_10_ helix within the EF loop at the C-tip is colored in orange. Cysteines and the exposed specificity residue (residue position 14 in M1, *cf*. **Fig 1B**) are shown in sticks in atom-specific colors. Cysteines are highlighted in yellow (with sulfhydryl groups oriented towards hydrophobic core), orange (with potential for disulfide bridge formation), and red (with sulfhydryl group surface exposed). Zoom-ins of the N-tip (upper inlet) and C-tip (lower inlet) areas highlight those residues that are involved in specific side-chain mediated hydrogen bonds. For reasons of clarity, only hydrogen bonds formed by side chain/side chain interactions are shown. Residues that are conserved among different M-band sequences are labeled in red.

The aim of this study was to unravel common and diverse individual structural signature patterns of the ten-fold array of identically folded Ig domains of the C-terminal M-band segment of titin. To achieve this goal, we have solved the crystal structures of five titin M-band domains: M1, M3, M4, M7, and M10. All five structures have been determined to a resolution of less than 2.1 Å, allowing structural interpretation at the atomic level **(Fig 2, Table 1)**. At this resolution, the estimated coordinate error is generally less than 0.2 Å [47]. The highest resolution of 0.96 Å has been achieved for the structure of the M7 Ig domain, allowing complete resolution of most non-hydrogen atoms at the atomic level **(S2 Fig).** The structure of the M4 domain was determined in two different crystal forms, with one showing a trimeric M4 domain assembly and the other revealing a disulfide-mediated dimeric state **(Tables 1 and 2**). For the M10 domain, there are multiple structures available, in the absence and presence of the obscurin domain 1 (Obs-1) and obscurin-like 1 domain 1 (Obsl1 (Ig1)), both of which bind M10 specifically [15,16,20]. Structures of identical M-band domains from different crystal conditions (M4, M10) and different ligand binding states (M10) superimpose with root mean squares (r.m.s.) deviations of 0.4–0.6 Å, demonstrating that the respective domains do not undergo any conformational changes under specific binding conditions. Prior to this work, NMR structures of two slightly different M5 domain constructs were published [18,19] and are included in our comparative analysis wherever feasible.

**Table 2:** M-band domain structural diversity.

	PDB ID	Resolution [Å]	pI^a^	Association (structure)^b^	No. Cys (all/accessible)^c^	Molecular specificity signatures
**M1**	2bk8	1.69	4.51	monomer	2/0	BC loop, potential for intramolecular SS bridge
**M3**	6hci	2.12	5.18	dimer of dimers	3/3	C’D loop, DE loop
**M4**	3qp3	2.00	7.35	trimer	3/1	
**M4**	6h4l	1.60		monomer	3/1	Metal-assisted intermolecular SS bridge
**M5**	1ncu	(NMR)	5.34	monomer	1/0	N/A
**M7**	3puc	0.96	8.73	monomer	2/0	DE loop
**M10**	3q5o	2.05	4.39	dimer	2/1	C’D loop, DE loop

a) pI, calculated isoelectric point (calculated from ProtParam [48])

b) *cf*. **S1 Fig**

c) *cf*. **Figs 1 and 2, S4 and S5 Tables**

As expected, all M-band domains investigated belong to the I-set Ig domain category, which is most common in muscle filament proteins [3,11,49,50] **(Fig 2).** I-set Ig domains contain a split β-strand A/A’, of which the first part A is associated with the β-sheet composed of strands ABDE and the second part A’ is associated with the opposite β-sheet composed of strands A’CC’FG. In addition to the ABDE/A’CC’FG β-sheet sandwich core, all M-band domain structures comprise two characteristic tip regions, which we refer to as “N-tip” and “C-tip” due to their proximity to the N-termini and C-termini of the respective domains **(Fig 2).** The N-tip is formed by three short loops. Two of them are intra-sheet loops connecting β-strands D and E (slightly below the tip), and F and G, originating from the two opposite sheets ABDE and A’CC’FG, respectively. The third loop connects β-strands B and C, originating from the two different β-sheets. The C-tip is solely formed by three sheet-connecting loops: A’B, C’D, and EF. The EF loop is the longest of all the tip loops and contains a highly conserved 3_10_ helix. The C’D loop, slightly below the very C-tip, is also relatively long and transverses across the surface of the ABDE β-sheet.

For the remaining four M-band domains (M2, M6, M8, M9), homology models were built using the most closely related Ig domain structures from the titin M-band segment **(S3 Fig and S2 Table)**. Most of these domains could be reliably modeled, with the exception of the I-set β-sheet association of the split β-strands A and A’ that is less robust in the respective models. In the absence of further experimental evidence, it is however unlikely that these M-band domains fall into a different Ig domain category other than the I-set observed in all experimental structures.

### Overall estimation of sequence and structure diversity in titin M-band domains

All experimentally determined M-band domain structures superimpose with r.m.s. deviations of 1.0 to 1.8 Å, except for the pairwise comparisons with the M5 domain that show significantly larger r.m.s. deviations of 2.2 to 2.3 Å **(Table 3),** due to the different experimental structural determination approach by NMR spectroscopy. Based on these data, for the available M-band domain X-ray structures (M1, M3, M4, M7, M10) the numerical structural diversity can be estimated in the order of 0.5–0.8 Å, after subtraction of the estimated coordinate error and conformational diversity of surface residues due to specific crystallization conditions. The calculated pairwise sequence identities of these M-band domains based on respective structure positions are in the range of 18% (M1/M5) to 30% (M4/M5), which is quite low in light of the relatively high level of structural conservation. When the high level of sequence identity of residues forming the hydrophobic core of the respective Ig domains is taken into consideration (details shown below), the sequence diversity of the surface of these M-band domains is even more pronounced. In line with these observations, we found no obvious correlation between the level of overall structural and sequence similarity in different M-band domain pairs **(Table 3)**. A phylogenetic analysis did not reveal any regular pattern of conservation that could provide clues about the evolvement of M-band domains, unlike other parts of titin [51].

**Table 3:** Structure-based sequence comparison *versus* structure comparison.

	Sequence identity [%]										
**Structural similarity [Å]**		M1	M2	M3	M4	*M5*	M6	M7	M8	M9	M10
	M1		26.1	21.6	27.3	*18*.*2*	19.8	19.3	20.9	23.9	24.4
	M2			30.3	31.9	*28*.*1*	27.6	**33.7**	19.5	27.0	25.8
	M3	1.80			27.0	*23*.*6*	18.2	28.1	19.5	**33.7**	24.1
	M4	1.44		1.17		*30*.*3*	19.5	23.6	**24.1**	29.2	25.3
	*M5*	*2*.*44*	* *	*2*.*32*	*2*.*26*	* *	*16*.*1*	*29*.*2*	*23*.*0*	*27*.*0*	*28*.*7*
	M6							**21.8**	18.8	18.4	24.1
	M7	1.54		1.31	1.01	*2*.*26*			19.5	27.0	27.6
	M8									24.1	22.4
	M9										28.7
	M10	1.54		1.48	1.07	*2*.*33*	* *	1.21			

M-band domains with structures determined are underlined (M5, NMR structure, in italics). Sequence identities of M-band domains used for generation of homology models of M2, M6, M8, and M9 are in bold. Structural similarities were calculated with PDBeFOLD (Protein structure comparison service PDBeFold at European Bioinformatics Institute; (http://www.ebi.ac.uk/msd-srv/ssm)). Sequence identity matrix was calculated with Clustal2.1 [45].

To identify structural signature features that may generate the distinct functional properties of individual M-band domains, we used three different approaches. Firstly, we analyzed the structural conservation of the cores of all M-band domains with known structures by directly comparing the underlying β-sheet structures. Next, we identified hot spots in terms of individual structural diversity by plotting pairwise spatial differences based on structural superimposition. Thirdly, we characterized conserved and divergent sequence features using a structure-based sequence alignment.

### The Ig β-sheet structure is highly conserved in titin M-band domains

**Fig 3:**
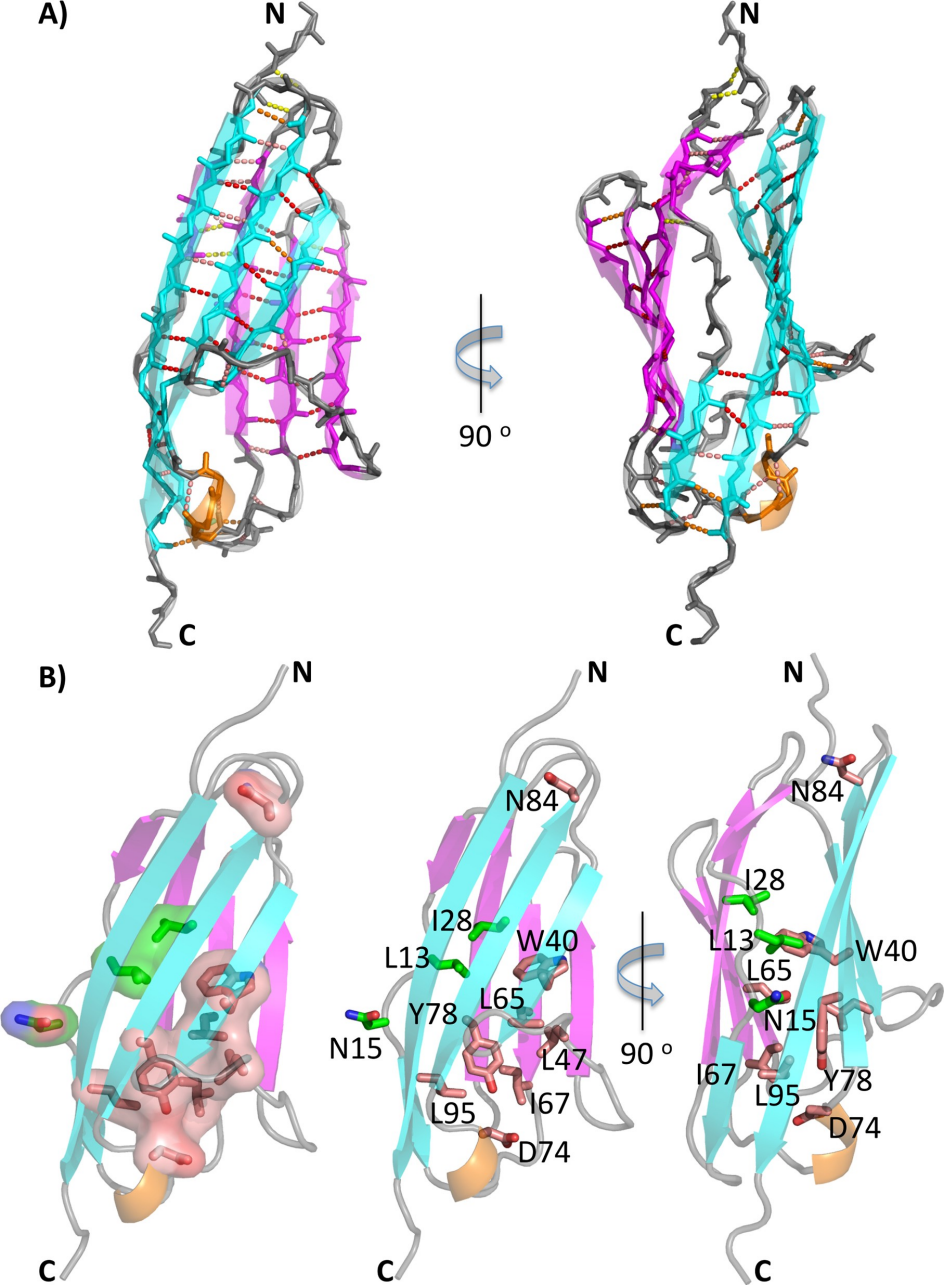
Conserved structural elements in M-band domains. A) Conservation of main chain hydrogen pattern: 6/6 structures, red; 5/6 structures, salmon; 4/6 structures, orange; 3/6 structures, yellow. The numbers of conserved hydrogen bonds are listed for the two β-sheets ABDE and A’CC’FG, and the C-tip area. B) Display of residue positions that are conserved among different M-band sequences (*cf***. Fig 1B**) in salmon, and residues that are conserved for each M-band domain among different species but variable in different M-band domains in green. From the left to right: semi-transparent surface presentation (left), stick representation (center), and stick representation rotated by 90° around a vertical axis (right). The color scheme is consistent with **Fig 2**.

Direct comparison of the β-sheet hydrogen bond pattern of the six M-band domain structures provides insights into the level of structural conservation. Both opposite β-sheets ABDE and A'CC'FG indeed comprise a highly conserved pattern with more than 20 specific hydrogen bonds per sheet **(Fig 3).** The majority of these are at structurally identical positions in five of the six available structures. As expected, diminished structural hydrogen bond pattern conservation is found in the peripheral areas of both sheets close to the N-tip and C-tip of the overall structure and in interactions involving the split β-strand A/A’. In conclusion, structural diversity in these M-band domains is restricted to the loop regions at the two tips, close to the N- and C-termini of each domain, whereas the core of the six M-band structures is highly conserved.

### Individual M-band domain signatures by structural comparison

Plots of structural deviations per residue along the different M-band domain crystal structure sequences, using the structures of M7 and M10 as reference, reveal three distinct segments **(Fig 1C, S4 Fig)**. The N-terminal split A/A’ β-strand segment shows a generally elevated level of spatial differences. This finding reflects the conformational variability observed for this segment in the respective crystal structures, in which the extent of the split A/A’ β-strand is considerably more variable than those of other secondary structural elements of the M-band domains **(Fig 2)**. The second segment in the pairwise analysis of spatial deviations ranges from the A’B loop to the DE loop. This region features several significant individual M-band domain deviations for M1 (BC loop), M3 (C’D loop, DE loop), and M7 (DE loop), indicating potential M-band domain signature motifs (discussed in more detail below). Finally, the C-terminal segment, ranging from the DE loop to the C-terminus, presents the most structurally conserved area. Taken together, the data demonstrate that potential signatures of individual M-band domains have specific localizations mostly in loop segments spanning over large parts of the respective Ig domain fold.

### Structural diversity in titin M-band domains

We have generated a structure-based sequence alignment from the superimposition of all M-band domains with known experimental structures **(Fig 1B).** For clarity, we have established revised numbering schemes for each M-band domain to facilitate direct comparison of structurally equivalent residue positions **(S3 Table, renumbered coordinate sets in Supplement),** rather than using residue numbers of the complete titin sequence (UniProt ID Q8WZ42). As there are deviations due to insertions/deletions within the structure-based alignment, we always quote the residue position of the M1 domain when discussing specific residues from other M-band domain structures. The alignment reveals six residue positions, which are identical both between different M-band domains and among different species for the same M-band domain **(Fig 1B, Fig 3B, S5 Fig),** whereas another five residue positions are highly conserved. Most of these residues are involved in forming a cluster as part of the hydrophobic core of each of these M-band domains, with a particularly strong contribution to the lower part close to the C-tip **(Fig 3B)**.

Within this core, Y77 (M1) from β-strand F **(Figs 2 and 3B)** has the specific role of generating an invariant hydrogen bond to the main chain carbonyl group of D73 (M1) at the conserved 3_10_ helix situated at the C-tip, connecting β-strands E and F from the two opposite β-sheets. In four of the six structures (M1, M4, M7, M10), D73 (M1), also invariant in all M-band sequences, forms an additional salt bridge with a conserved lysine/arginine (K52 in M1), located at the CD loop that connects two β-strands from the two opposite β-sheets **(Fig 2)**. In the M5 domain NMR structure, a defined interaction of this residue pair is missing, probably due to a lack of experimental data supporting the interaction. The conserved lysine/arginine position in the M3 domain is replaced by an asparagine (N52 in M3, K52 in M1), and hence no interaction with the equivalent aspartate (D74 in M3, D73 in M1) is found. As N52 in M3 is close to an M3 structural motif that significantly deviates from the other structures **(Fig 1C),** it is plausible that the loss of this specific interaction is of functional relevance and part of a specific M3 signature. These conserved C-tip interactions are embedded in various non-conserved interactions, adding structural diversity to the C-tip. Direct comparison of the C-tip areas of all six M-band structures also reveals substantial differences in the interaction network complexity, which is particularly high in the structures of M4, M7, and M10 **(Fig 2)**.

The invariant residue N83 (M1) is located at the N-tip GH loop and so is structurally separate from the other invariant residues in the M-band domains **(Figs 2 and 3B)**. This asparagine connects the two intra-sheet loops BC and GH via several hydrogen bonds in all M-band domain structures apart from M1, and thus seems to be crucial for establishing a conserved and compact N-tip structure. In the M1 domain, the BC loop has a distinct sequence composition and structure that significantly differs from the other M-band domains **(Figs 1C and 2A).** In this structure, no residues from this loop interact with N83 (M1), possibly establishing an M1 domain-specific signature.

**Fig 4:**
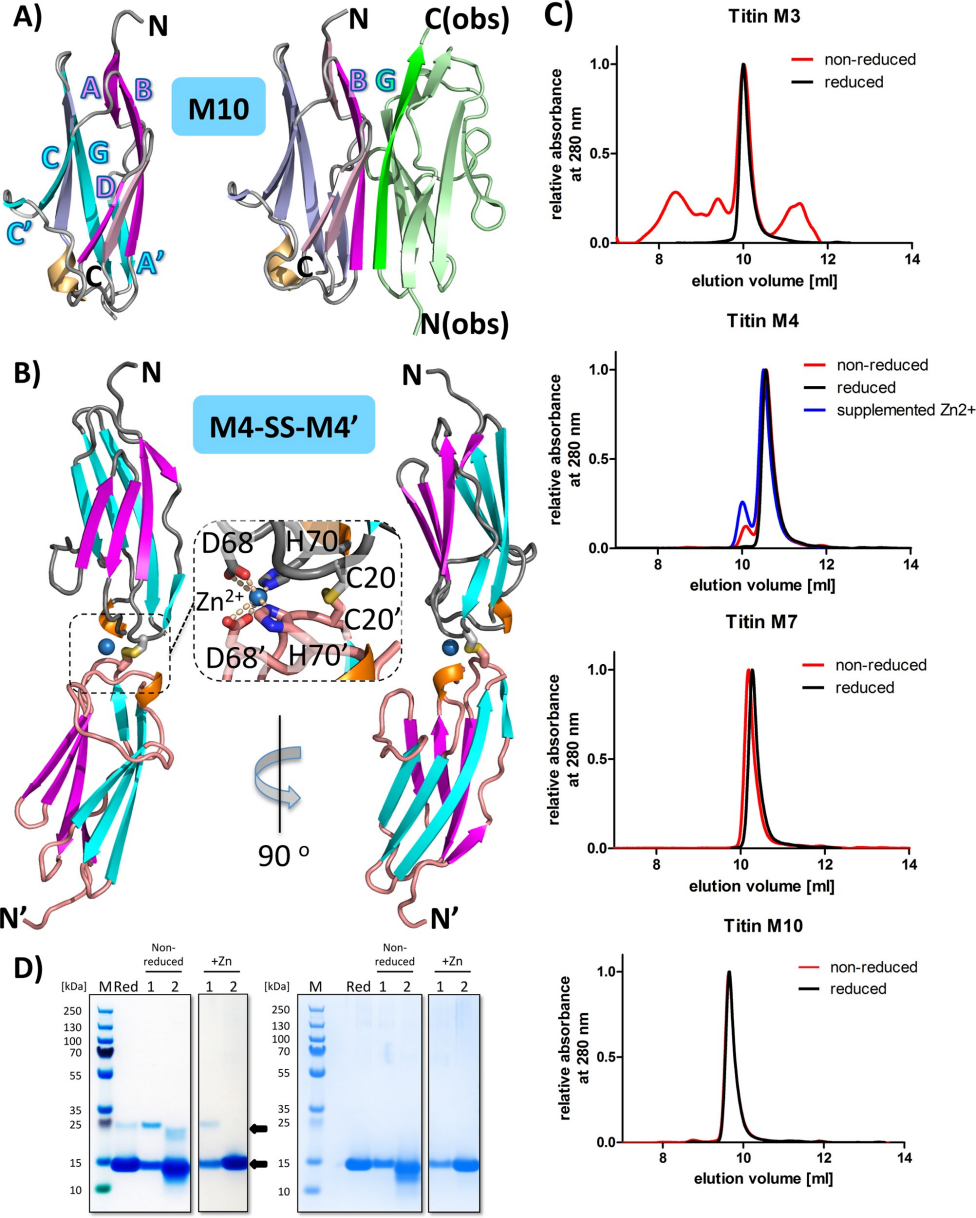
Mechanisms of M-band domain assembly. A) Structures of apo-M10 and M10 in complex with obscurin [20]. In the apo-M10 structure, β-strands with unused valences of hydrogen bond-mediated β-sheet formation are shown in strong colors and are labeled. The remaining β-strands are shown in paler colors. In the structure of the M10-obscurin complex, only β-strand B, interacting with β-strand G of obscurin (green), is shown in strong color (magenta). Other parts of the obscurin molecule are shown in pale green. B) Structure of the M4-SS-M4’ assembly. Colors are as in **Fig 2,** expect that the loops of the second M4’ molecule are shown in light pink. The zoomed-in inset shows structural details of the M4-M4’ disulfide bridge and the Zn^2+^ ion (blue sphere), which is symmetrically bound in a tetrahedral arrangement by two aspartates (D68, D68’) and two histidines (H70, H70’) from M4 and M4’. C) Superimposed and normalized gel filtration elution curves of M1, M3, M4, and M7 under reducing (green) and non-reducing (violet) conditions. For the M4 domain, in addition, the elution curve under non-reducing conditions in the presence of Zn^2+^ is shown (pale violet). D) Non-reducing (left) and reducing (right) SDS-PAGE performed for all samples obtained during purification of the M4 domain as shown in Fig 4C, with M (marker), Red (sample purified under reducing conditions), numbers 1 and 2 indicating the early (1) and the late (2) peak during elution of the purification under non-reducing conditions, and supplementation of Zn^2+^.This indicates limited disulfide bridge-mediated covalent assembly under non-reducing conditions, as indicated by arrows. This effect is enhanced in the presence of Zn^2+^. SDS-PAGE gels of the all M-band domains investigated are shown in **S6 Fig**.

The structure-based alignment has also identified three residues that are distinct in different M-band domains but invariant among different species for each individual M-band domain **(Figs 1B and 3B, S5 Fig)**. Hence, these residues are likely to contribute to specific structural and functional signature patterns across different M-band domains. Two of them are located at an exposed loop, connecting the two split β-strands A and A’ (positions 12 and 14 in M1); the third one is located at β-strand B (position 27 in M1). The side chain of residue 14 in particular is highly exposed in all structures and thus likely to contribute to specific structural and functional signatures in all M-band domains. This proposal is supported by known structures of the M10 domain with Obs-1 or Obsl1 (Ig1), in which the equivalent residue D15 (H14 in M1) is close to a major interaction site in the respective protein/protein interface [15,20] **(Fig 4A)**.

The sequences of the six M-band domain structures investigated have one to three cysteines each. Our structure-based alignment, however, shows no indication of a conserved cysteine pattern **(Fig 1B)**. Most cysteines are oriented towards the hydrophobic core and are solvent inaccessible **(Fig 2, Table 2).** By contrast, the three cysteines of the M3 domain (C18, C25, C91, equivalent to G17, K24, S90 in M1), which are located in different β-strands A’, B, and G, are all at least partially surface exposed **(S4 Table).** Two other M-band domains each contain a single exposed cysteine: C20 in the M4 domain (E19 in M1) located at the A’B loop at the C-tip, and C42 in the M10 domain (F42 in M1) located at the tip of the CC’ loop near the C-tip.

In the M1 domain, we found the only case where two cysteines–C27 from β-strand B and C79 from β-strand F–in the hydrophobic core are properly positioned for potential disulfide bridge formation, connecting the two opposite β-sheets ABDE and A'CC'FG **(Fig 2)**. The distance of the sulfhydryl groups of the respective face/face cysteines is 4.0 Å. Interestingly, the homologous models of the domains M6 and M8 are suggestive of two further cases of potential disulfide bridge formation **(S3 Fig),** one providing another connection between the two opposite β-sheets (M6) and the other establishing an additional connection within one of the two β-sheets (M8). Taken together, the non-conserved cysteine pattern in the structures of the M-band domains could generate further functional diversity among different M-band domains with respect to specific redox conditions and potential post-translational modifications such as S-glutathionylation [52,53].

### Limited redox-regulated M-band domain assembly

Many Ig domains from filamentous, multi-domain proteins, including titin, are known for their unique capacity to form either specific non-covalent self-assemblies or heterodimeric assemblies with other Ig domains [54]. However, except for the established β-sheet-mediated interaction of the C-terminal M10 domain with both Obs-1 and Obsl1 (Ig1) [15,16,20], no further β-sheet interactions by any other titin M-band domains have been characterized to date. In the structural titin M-band domain repository presented here, we found three cases for M-band domain self-assembly in domains M3, M4, and M10, irrespective of crystal symmetry, which is not taken into further consideration in this contribution as in solution these domains are mainly monomeric **(Tables 1 and 2, S1 Fig)**.

An alternative way of covalent Ig domain assembly under non-reducing conditions may be via intermolecular disulfide bridge formation involving surface-exposed cysteines. For titin, such potential has been shown for several sections of the entire sequence under *in vitro* conditions but the M-band segment has not yet been investigated in detail [55–58]. Based on our analysis of cysteine accessibilities **(Table 2, S4 Table),** domains M3, M4, and M10 are candidates for such linkages. We validated our structural findings by measuring the free thiol content for domains M3, M4 and M10 by titrating them with 5,5'-dithiobis-(2-nitrobenzoic acid) (DTNB), using M7 (which has two buried cysteines only) as a negative control. As expected, we found one free cysteine equivalent for domains M4 and M10, and almost three free cysteine equivalents for M3 **(S5 Table)**.

In one of the two crystal structures of the M4 domain, we found evidence for an intermolecular disulfide bridge across a crystallographic two-fold axis. It is mediated by two exposed cysteines, both C20 from two M4 domains at the C-tip coupled with an intermolecular Zn^2+^-binding site with tetragonal association of D68 (D68 in M1) and H70 (T70 in M1) from the two interacting M4 domains **(Fig 4B)**. This interaction generates an elongated C-tip/C-tip M4-SS-M4’ complex. This result is in line with our cysteine surface accessibility analysis, showing that C20 of the M4 domain is the most exposed (92% surface accessibility) of all M-band domain cysteines analyzed **(S4 Table)**.

Based on these structural findings, we also analyzed the potential for self-assembly of the three M-band domains with exposed cysteines M3, M4 and M10, again using M7 as a negative control. Under reducing conditions, all of the M-band domains tested were monomeric **(Fig 4C, S6 Fig),** rendering it unlikely that any of these domains play a role in longitudinal titin self-assembly. The data also indicate that the non-covalent assemblies found in the crystal structures of M3, M4, and M10 **(S1 Fig)** are most likely artifacts due to specific crystallization conditions. Nevertheless, they underline the potential of β-strands with unused valences for hydrogen-bond intermolecular-mediated β-sheet formation to form assemblies with other protein-binding partners.

Under non-reducing conditions, as expected, the M7 domain remained entirely monomeric **(Fig 4C, S6 Fig).** By contrast, for domain M4, we found an additional higher molecular peak when using size exclusion chromatography, indicating limited dimerization. Interestingly in the presence of Zn^2+^, which is found in the structure of the disulfide bridge-mediated M4 tandem arrangement **(Fig 4B)**, we detected a further increased level of M4 domain oligomerization **(Fig 4B–4D).** In correlation with our size exclusion data, SDS-PAGE analysis under reducing and non-reducing conditions revealed a covalent higher molecular weight species, indicating intermolecular disulfide bridge formation **(Fig 4C, S6 Fig)**. For the M10 domain, a residual higher molecular peak could be detected by size exclusion chromatography, but this was not detectable by SDS-PAGE analysis. Unlike our findings for M4 and M10, size exclusion chromatography of the M3 domain under non-reducing conditions revealed a mixture of different higher molecular weight species. We attribute this to possible combinatorial linkages through all three exposed cysteine residues under the given experimental conditions **(Fig 4C)**. In the corresponding SDS-PAGE analysis, higher molecular weight species were only faintly detectable **(S6 Fig)**. Taken together, our data indicate a significant potential for intermolecular disulfide bridge formation, especially in domains M3 and M4, with one or more highly exposed cysteines in the respective structures. Whether the potential to link to other domains with exposed cysteines takes place under physiological conditions within the M-band region of muscle sarcomeres remains a highly interesting topic for future research.

## Discussion

Long filament proteins of muscle sarcomeres comprise extensive arrays of identically folded domains, which we refer to as “beads on a string”. One of the main challenges in understanding the structural and ultra-structural organization of muscle sarcomeres is how these domains have developed an individual structural and functional profile at the single domain level. Indeed, how false-positive functional readouts from identically folded neighboring domains can be avoided has not been thoroughly investigated to date. Here, we focus on the C-terminal M-band segment of titin that harbors an array of ten Ig domains and establish an atomic resolution structural repository of more than half of these domains. Our data allow general principles of both structural domain conservation and diversity to be dissected. We found structural diversity hotspots in three exposed loops, with two at the N-terminal tip (loops BC and DE) and the third located at the C-terminal tip (loop C’D) **(Fig 1C)**. In addition, the split β-strand AA’, which is associated with both Ig fold β-sheets in all M-band structures determined, shows diversity in terms of the conformations observed **(Fig 2)**. Where structural variability is detected, it is frequently reflected in a distinct sequence pattern when compared with those of other M-band domains **(Fig 1B)**.

Many of the structural properties shared by the M-band Ig domains investigated here present general features found in the I-set of the Ig superfamily [50]. Examples are the conserved tryptophan in β-strand C (W40 in M1) and the highly conserved “tyrosine corner” in β-strand F (Y77 in M1), presenting two key residues for the stability and folding of I-set Ig domains **(Figs 1B, 2 and 3)** [59–61]. Other signature residues such as the conserved leucine in β-strand E (L65 in M1) are present in all M-band domain structures, with the exception of the M5 domain where there is a phenylalanine instead. A conserved leucine residue in β-strand G (L94 in M1) is also found in all M-band structures investigated, with the exception of M10 where this position is replaced by an isoleucine.

Structural data for an increasing number of Ig-like domains from other parts of titin are also presenting a rich repository for comparative analysis. As part of a structural investigation of repetitive arrangements of Ig domains from the titin I-band region, a “N-conserved type” was identified showing correlated occurrence of three conserved features within the N-tip region: a PP motif within β-strand A, a PxP/PPx motif within the BC loop and a NxxG motif within the FG loop [62]. Whereas the latter FG loop motif is found in all M-band domains investigated here and is virtually invariant among the sequences from different species, the other two less conserved motifs are found at least in part in five M-band domains (M3, M4, M5, M7, M10) but not in domain M1 (**Figs 1B and S5**). Our data, together with those previously published [62], reemphasize the significance of the distinct structural conformation of the N-tip region in domain M1, most strongly featured by the presence of an unrelated BC loop motif **(Figs 1C, 2A and S4)**. The C-tip region of all M-band domains investigated follows previous findings in terms of sequence and structure for other titin Ig domains characterized. Whereas the 3_10_ helix-containing EF loop is comprised by a DxGxY motif that is highly conserved in other titin Ig domains [12], in contrast, the C’D loop is generally highly variable except one conserved leucine/isoleucine position at the C-terminus of β-strand C’ that plays a crucial role in fold stabilization (**Fig 1B**) [63]. In our analysis of titin M-band domains, the C’D loops of M3 and M10 are flagged for being most diverse **(Fig 1C)**.

Another key contributor to structural diversity is in the non-conserved distribution of highly exposed cysteines, which are of specific interest as they are highly reactive, polarizable, and redox-active in response to specific physiological conditions such as pH and redox environment [64]. Hence, the presence of exposed cysteines could link specific M-band domains to other filament proteins via intermolecular disulfide bridges under favorable non-reducing conditions. We show that two M-band domains with exposed cysteines (M3, M4) have limited potential for disulfide bridge-mediated linkage as purified proteins, additionally supported by a structure of a disulfide bridge-mediated M4 domain dimer (M4-SS-M4’) **(Fig 4B).** Our structural data mirror an earlier notion that cysteines at positions conserved in many other titin Ig domains on β-strands B, F, and G, are less frequently found in M-band domains but are complemented by various additional non-conserved cysteine positions **(Fig 1B)** [55]. Finally, in reflection to the diversity of linkers connecting neighboring M-band domains, we could not find any specific sequence and structural patterns at the N- and C-termini of all M-band domain structures investigated, contrasting previous observations on conserved structure and sequence motives observed in structural analyses of multi-domain arrangements in the I-band and A-band regions of titin where extensive Ig domain patterns with super-repeat topology are found [14,62,65].

Available structural data on functional titin M-band domain assemblies with other filament proteins are limited to structures of the C-terminal M10 domain with the N-terminus of another long filament protein Obs and Obsl1. Therefore, we used those as a test case to identify possible molecular parameters for M10 functional specificity [15,16,20] **(Fig 4)**. In the M10–Obs/Obsl1 assembly, an extensive intermolecular β-sheet is formed by M10 β-strand B and obscurin/Obsl1 β-strand G. However, as these β-strands are structurally highly conserved **(Figs 2 and 3)** and therefore do not explain the lack of binding with other M-band Ig domains, we focused instead on amino acid-specific interactions observed in the corresponding interface. We identified three residues located at M10 domain β-strand B–T25, A27, A29 (K24, V26, K28 in M1)–and one on β-strand A (A11, Q11 in M1) that are all highly exposed in the separate M10 domain but buried in the M10–Obs-1/Obsl1 (Ig1) complex **(Fig 4)**. Two of these residues (T25, A27) were previously shown to substantially reduce binding affinity to both Obs-1 and Obsl1 (Ig1) [16], suggesting that combining mutations in these residues would lead to diminished or loss of binding. As shown in the corresponding structure-based sequence alignment **(Fig 1B)**, these four residue positions are replaced almost exclusively by large and hydrophilic amino acids in other M-band domains, suggesting that these cumulative changes are critical for avoiding unwanted binding to Obs-1/Obsl1 (Ig1) by other M-band domains except for M10.

Although there has been a substantial increase in the knowledge of C-terminal titin M-band section *TTN* variants [66], some of the most interesting variants associated with hereditary muscle diseases are not yet curated in relevant databases such as the Exome Aggregation Consortium (ExAC) [67], which allows direct comparison by applying objective significance criteria. In many cases, identified variants on their own are not sufficient to cause a specific disease phenotype, which hence may require additional mutations that are however not known or not further characterized.

Genetically, the majority of identified M-band *TTN* disease-associated variants lead to truncated titin fragments and hence a straightforward effect on the functional readout in the associated phenotype as a result of the loss of specific domains. For *TTN* M-band variants that lead to mutated, non-truncated titin polypeptide chains, structure-based interpretation to elucidate genotype/phenotype relationships remains an attractive option. Attempts have been made to model such mutations systematically [68], to biophysically characterize them [69], and to interpret them based on experimental structural data [15,16,20]. Conclusive structure-based interpretations, leading to either loss-of-function effect with the structure remaining intact or affecting the structure itself with subsequent functional effects, however, have remained scarce.

In light of these existing complexities, we limit our analysis to those functionally annotated *TTN* variants, either in the ExAC database or recent publications [66,69,70] (**Table 4).** For potential functional impact, we have analyzed i) the role of the residue within the structure, with possible structural consequences of the mutation; ii) the involvement in known binding sites to the applicable extent; iii) the conservation of the residue positions within sequences across different species and across different M-band domains **(Fig 1B).** Based on these criteria, there are three *TTN* variant residue positions all found in the M10 domain, which are likely to have major structural/functional effects. Two of them (W40R and L66P, equivalent to W40 and L65 in M1) belong to the conserved residue cluster that forms a common hydrophobic core in all M-band domains with known structures **(Fig 3B).** The third M10 variant is associated with a cysteine (C28Y, C28S, equivalent to C27 in M1) that points towards the hydrophobic core of M10 **(Fig 2F).** As described above, two of its adjacent neighbors (T25, A27) are directly implicated in Obs-1/Obsl1 (Ig1) binding.

**Table 4:** Disease-associated mutations.

	UniProt	Residue	Mean allele frequency	Predicted stability ^a^	Reference ^b^	Sequence conservation ^c^	Structure	Predicted Structural Impact
**M1**								
G->R	32510	21	8.28E-06	-0.638	rs191522469		Loop	Altered loop conformation
I->V	32558	69	0.004253	-1.471	rs56347248		Hydrophobic core	
**M3**								
E->Q	32742	27	0.003337	-0.821	rs148525155		Surface	
I->N	32770	55	8.3E-06	-2.402	rs72629784		Hydrophobic core	Fold destabilization
T->M	32790	75	0.0006962	0.153	rs192001910		Loop	
**M4**								
G->D	33315	21	8.28E-06	-3.034	rs145748940	C	Loop	Altered loop conformation
H->Y	33364	70	8.29E-06	0.777	rs116876353		Loop	
T->M	33387	93	8.29E-06	-0.162	rs56001826		Surface	
D->E	33389	95	8.28E-06	-0.179	rs72629789		Surface	
**M5**								
V->M	33536	54	0.00308	-0.711	rs55865284		Hydrophobic core	Fold destabilization
K->Q	33568	86	1.66E-05	0.167	rs56365812	S	β-strand, exposed	
**M10**								
C->S	34277	28	8.45E-06	-1.373	rs193212275	S	Hydrophobic core	
C->Y	34277	28	0.000101	-1.373	rs193212275	S	Hydrophobic core	Fold destabilization
E->Y	34286	37	NA	NA	[71]		β-strand, exposed	
V->K	34287	38	NA	NA	[71]		Hydrophobic core	
T->E	34288	39	NA	NA	[71]		β-strand, exposed	
W->K	34289	40	NA	NA	[71]	C	Hydrophobic core	
W->R	34289	40	NA	-2.172	[70,72]	C	Hydrophobic core	Fold destabilization
H->P	34305	56	NA	-0.76	[69,70,73]		Loop	Altered loop conformation
I->N	34306	57	NA	-1.845	[69,70,74,75]		Hydrophobic core	Fold destabilization
L->P	34315	66	NA	-1.745	[70]	C	Hydrophobic core	Fold destabilization

Single Nucleotide Polymorphisms (SNPs) were extracted from Exome Aggregation Consortium (ExAC) database server [67] or, from referenced publications.

^**a**^ Predicted stabilization (kCal mol^-1^) extracted from TITINdb [68] using DUET server [76]. Negative values indicate a destabilizing effect.

^b^ Reference SNP ID number, or “rs” ID, [77]

^c^ C, conserved; S, specific; for further details *cf*. **Fig 1B**

Taken together, the analysis demonstrates that a large-scale structural repository is useful for interpretation of available genetic data of disease-associated titin variants leading to distinct structural alterations at the level of individual domains. This study exemplifies the potential of high-resolution structural biology to assign individual structural/functional properties to–at low resolution and the level of structural predictions–identically looking protein domains. Continuing such an approach for other segments of titin and other protein filaments with related domain structures could assist in systematic mapping of specific functions and protein/protein interactions. This would allow alterations to be tested either by exploiting information from clinical variants or by using designed single-residue mutations. Ultimately, this will help to close the gap between high-resolution molecular structures and cellular data so that we can more fully understand the highly crowded and interconnected environment found in muscle sarcomeres.

## Supporting information

S1 Fig M-band domain assemblies found in crystal structures (*cf*. Tables 1 and 2). Color codes: chain A, pale green; chain B, pale cyan; chain C, pale pink; chain D, pale yellow. Where intermolecular β-sheet formation is involved, the interacting β-strands are colored in red and labeled.
(TIF)

S2 Fig Representative 2*F*_o_−*F*_c_ electron-density map (blue) of the M7 domain structure drawn at 2σ contour level. (TIF)

S3 Fig Cartoons of structural homology models of M-band domains M2, M6, M8 and M9. Presentations are as in Fig 2. No zooms into the N-tip and C-tip areas are shown, due to lack of precision of modeled coordinates. For further statistics on homology models, see **S2 Table**.
(TIF)

S4 Fig Plot of spatial differences for residue pairs of available M-band X-ray structures, using the structure of M10 as reference. Color codes: M1, yellow; M3, orange; M4, magenta; M7, blue. The positions of secondary structural elements and sequence numbers (***cf*. S3 Table)** are indicated.
(TIF)

S5 Fig Conservation of M-band domains across different species. The alignment has been taken from **Fig 1B** and indicates the level of sequence conservation among sequences of the same M-band domains from different species. Color codes: orange, invariant; yellow, conserved. Other graphical elements have been adopted from **Fig 1B**.
(TIF)

S6 Fig SDS-PAGE gels for M-band domains M3, M4, M7 and M10, both under reducing and non-reducing conditions. Row annotations 1 and 2 refer to dimeric peak (1) and monomeric peak (2). Data for M4 has already been shown in Fig 4.
(TIF)

S1 Table Titin M-band constructs expressed in *E*. *coli*. (DOCX)

S2 Table M-band domain homology models. Homology models were calculated using SWISS-MODEL protein structure homology-modeling server [44]. Sequence similarity, Sequence coverage and Global Model Quality Estimate (GMQE) were calculated by the SWISS-MODEL server.
(DOCX)

S3 Table M-band domain residue renumbering. Where multiple protein chains are found, chain A was used.
(DOCX)

S4 Table Quantitative analysis of cysteine surface accessibility. Maximum accessible surface area of cysteine side chain: 46.6 Å^2^. Cysteine surface accessibility was calculated using pymol (The PyMOL Molecular Graphics System, Version 2.0 Schrödinger, LLC.). Where applicable, mean and S.D. were calculated with program Excel (Microsoft Corporation, Redmond, WA) from different domain copies available in the pdb file (M3: four copies; M4: three copies; M10: two copies).
(DOCX)

S5 Table Free thiol titrations. Mean and standard deviations of the free thiol titrations were calculated with program Excel (Microsoft Corporation, Redmond, WA) from three independent repeats of an experiment.
(DOCX)

S1 Coordinate File Titin domain M1 (2bk8). (PDB)

S2 Coordinate File Titin domain M1 (2bk8), renumbered. (PDB)

S3 Coordinate File Titin domain M3 (6hci). (PDB)

S4 Coordinate File Titin domain M3 (6hci), renumbered. (PDB)

S5 Coordinate File Titin domain M4 (3qp3). (PDB)

S6 Coordinate File Titin domain M4 (3qp3), renumbered. (PDB)

S7 Coordinate File Titin domain M4 (6h4l). (PDB)

S8 Coordinate File Titin domain M4 (6h4l), renumbered. (PDB)

S9 Coordinate File Titin domain M7 (3puc). (PDB)

S10 Coordinate File Titin domain M10 (3q5o). (PDB)

S11 Coordinate File Titin domain M10 (3q5o), renumbered. (PDB)

S12 Coordinate File Titin domain M5 (1nuc), renumbered. (PDB)

S1 Validation Validation report titin M1 (2bk8). (PDF)

S2 Validation Validation report titin M3 (6hci). (PDF)

S3 Validation Validation report titin M4 (3qp3). (PDF)

S4 Validation Validation report titin M4 (6h4l). (PDF)

S5 Validation Validation report titin M7 (3puc). (PDF)

S6 Validation Validation report titin M10 (3p5o). (PDF)
