# Supplementary figures and images for: Structural diversity in the atomic resolution 3D fingerprint of the titin M-band segment
Source: PLoS One. 2019 Dec 19;14(12):e0226693. doi: 10.1371/journal.pone.0226693 (PMC6922384; doi:10.1371/journal.pone.0226693)

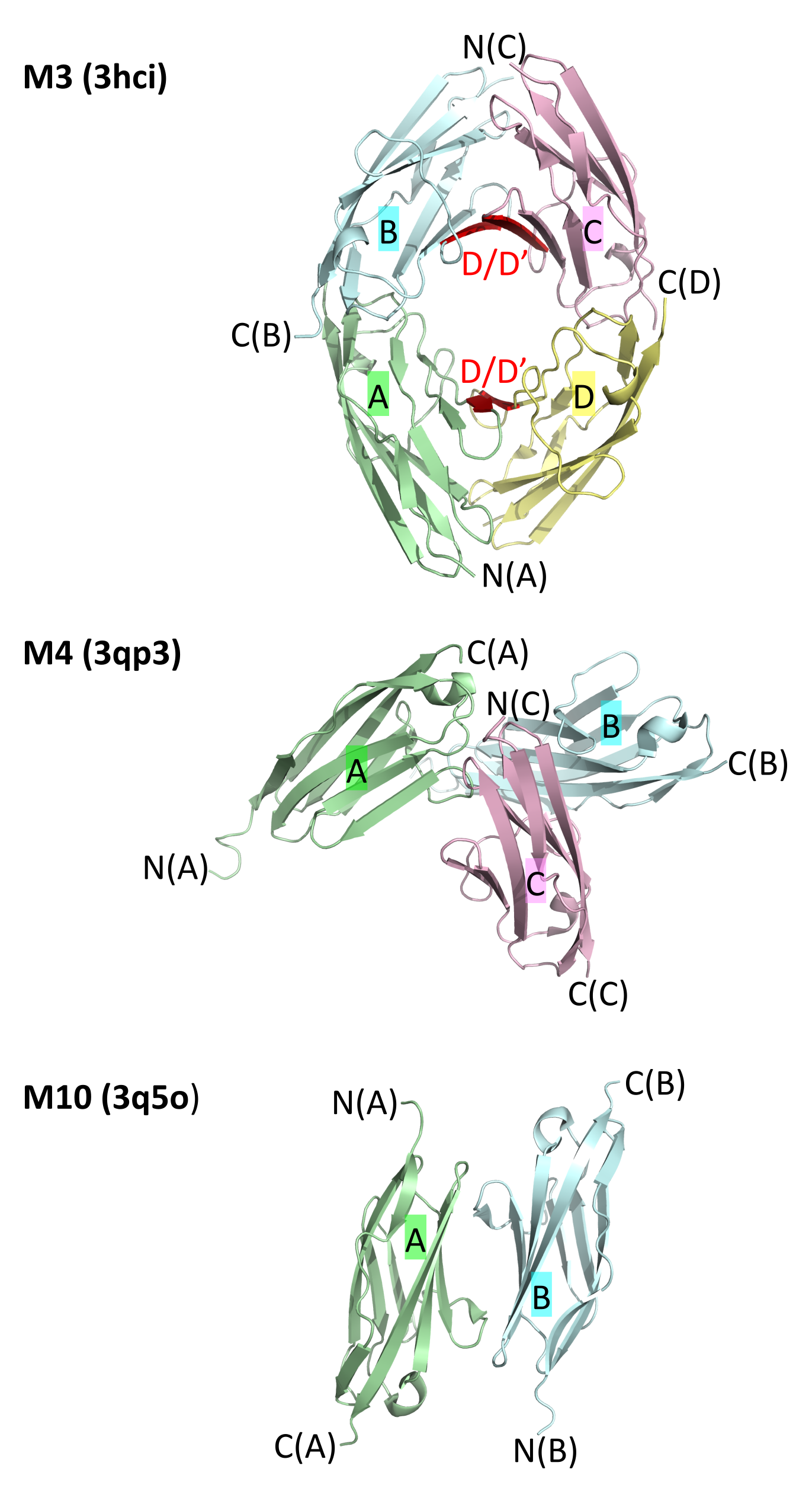

Supplement: S1 Fig — Color codes: chain A, pale green; chain B, pale cyan; chain C, pale pink; chain D, pale yellow. Where intermolecular β-sheet formation is involved, the interacting β-strands are colored in red and labeled. (TIF) [file pone.0226693.s001.tif]

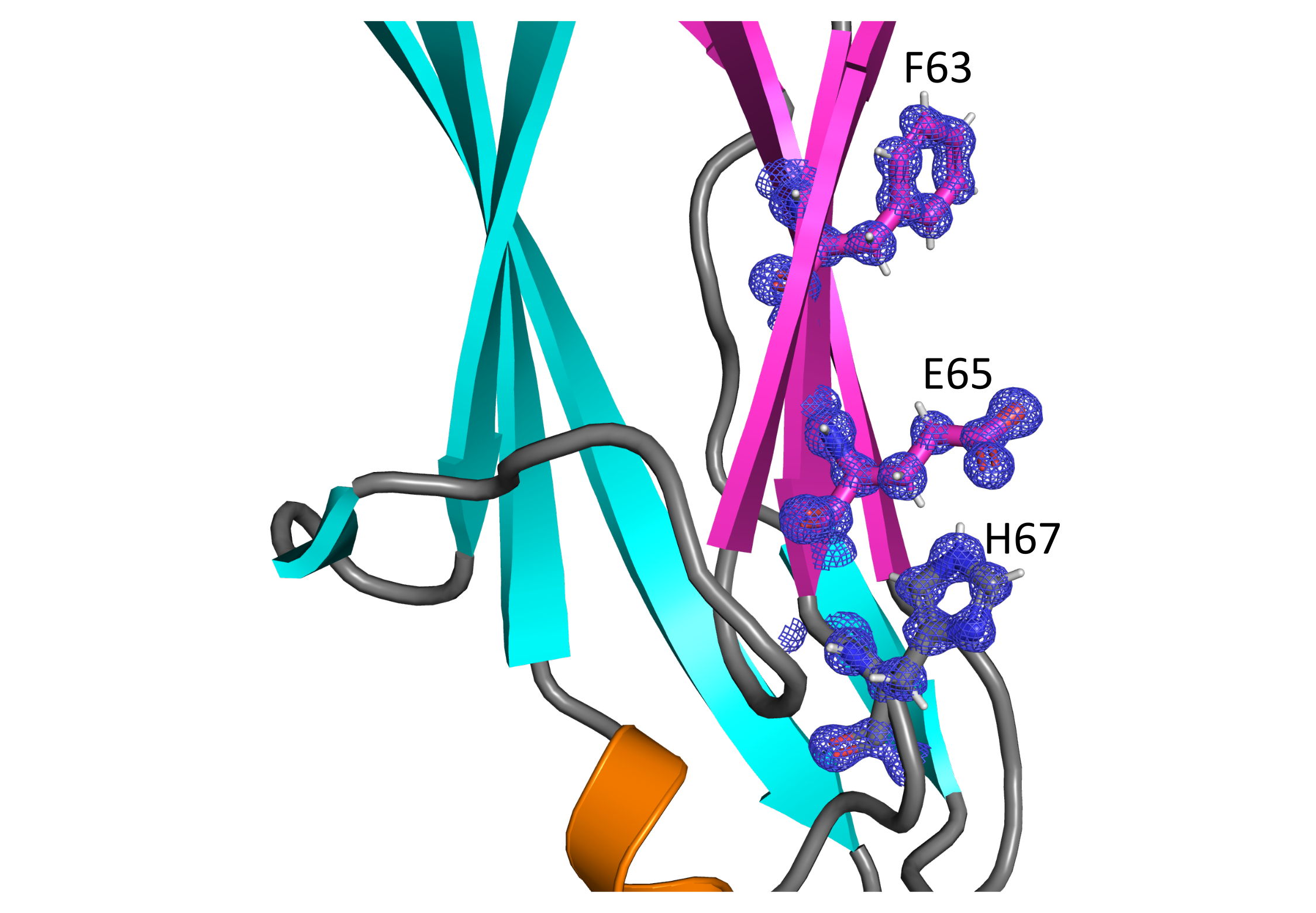

Supplement: S2 Fig — (TIF) [file pone.0226693.s002.tif]

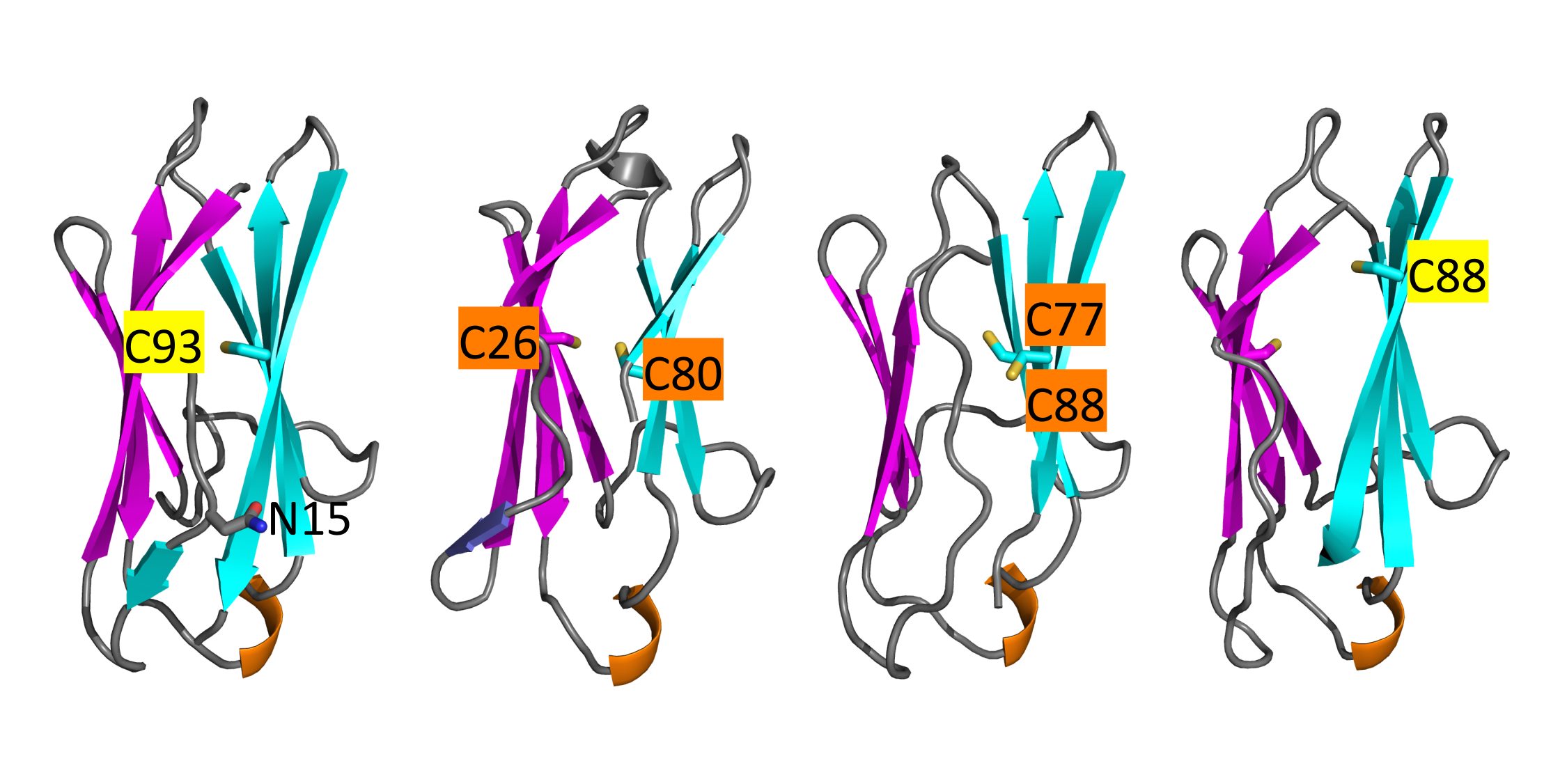

Supplement: S3 Fig — Presentations are as in Fig 2. No zooms into the N-tip and C-tip areas are shown, due to lack of precision of modeled coordinates. For further statistics on homology models, see S2 Table. (TIF) [file pone.0226693.s003.tif]

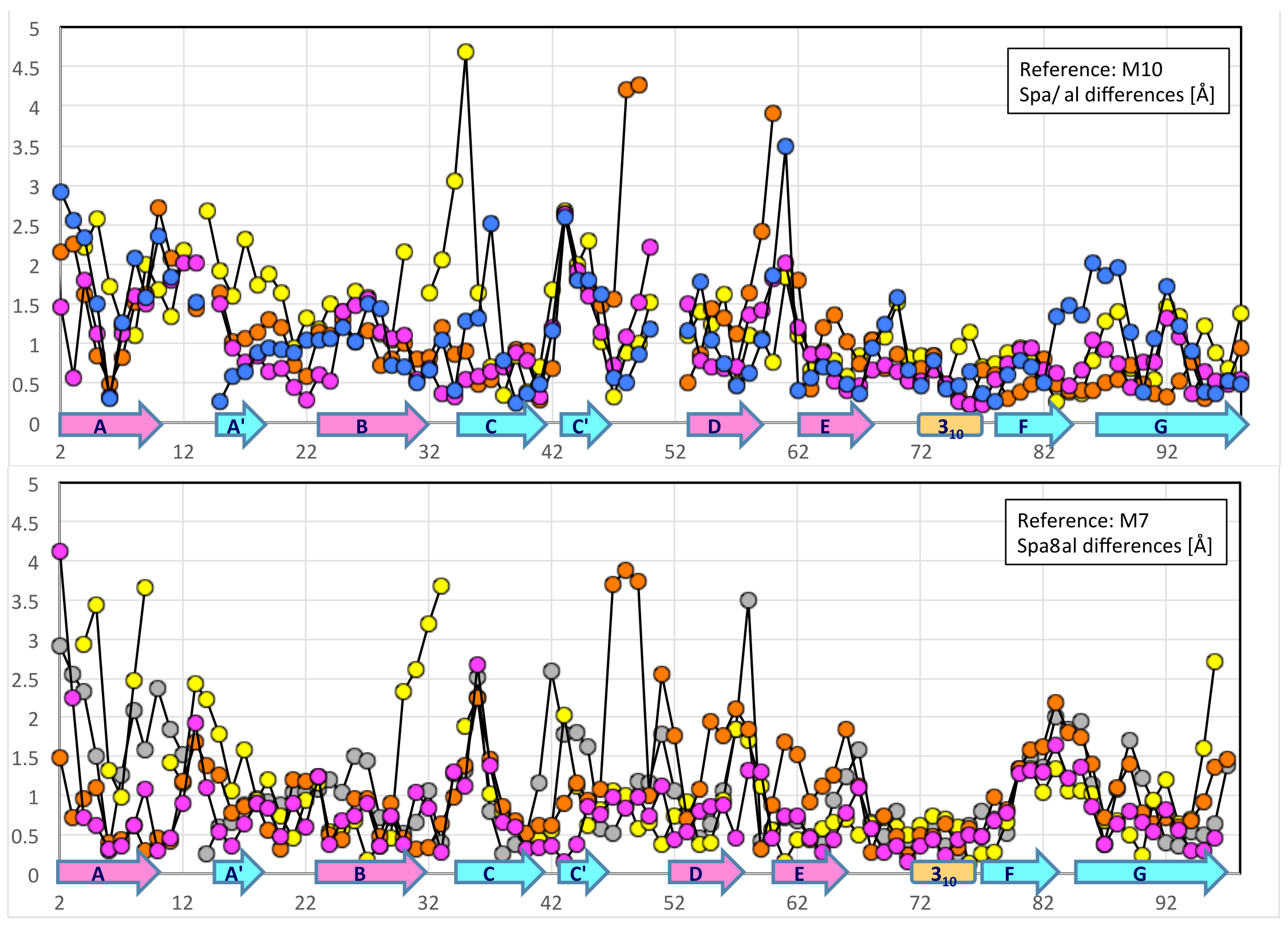

Supplement: S4 Fig — Color codes: M1, yellow; M3, orange; M4, magenta; M7, blue. The positions of secondary structural elements and sequence numbers (cf. S3 Table) are indicated. (TIF) [file pone.0226693.s004.tif]

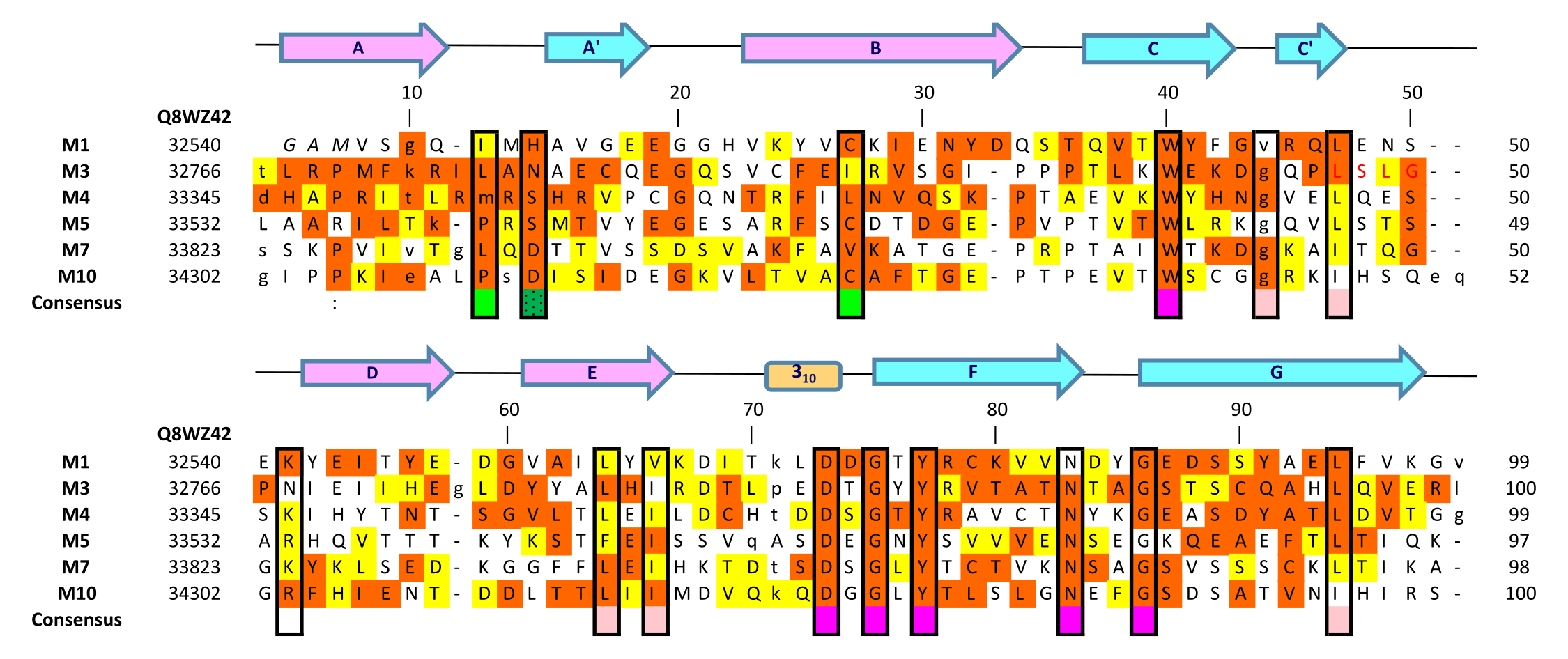

Supplement: S5 Fig — The alignment has been taken from Fig 1B and indicates the level of sequence conservation among sequences of the same M-band domains from different species. Color codes: orange, invariant; yellow, conserved. Other graphical elements have been adopted from Fig 1B. (TIF) [file pone.0226693.s005.tif]

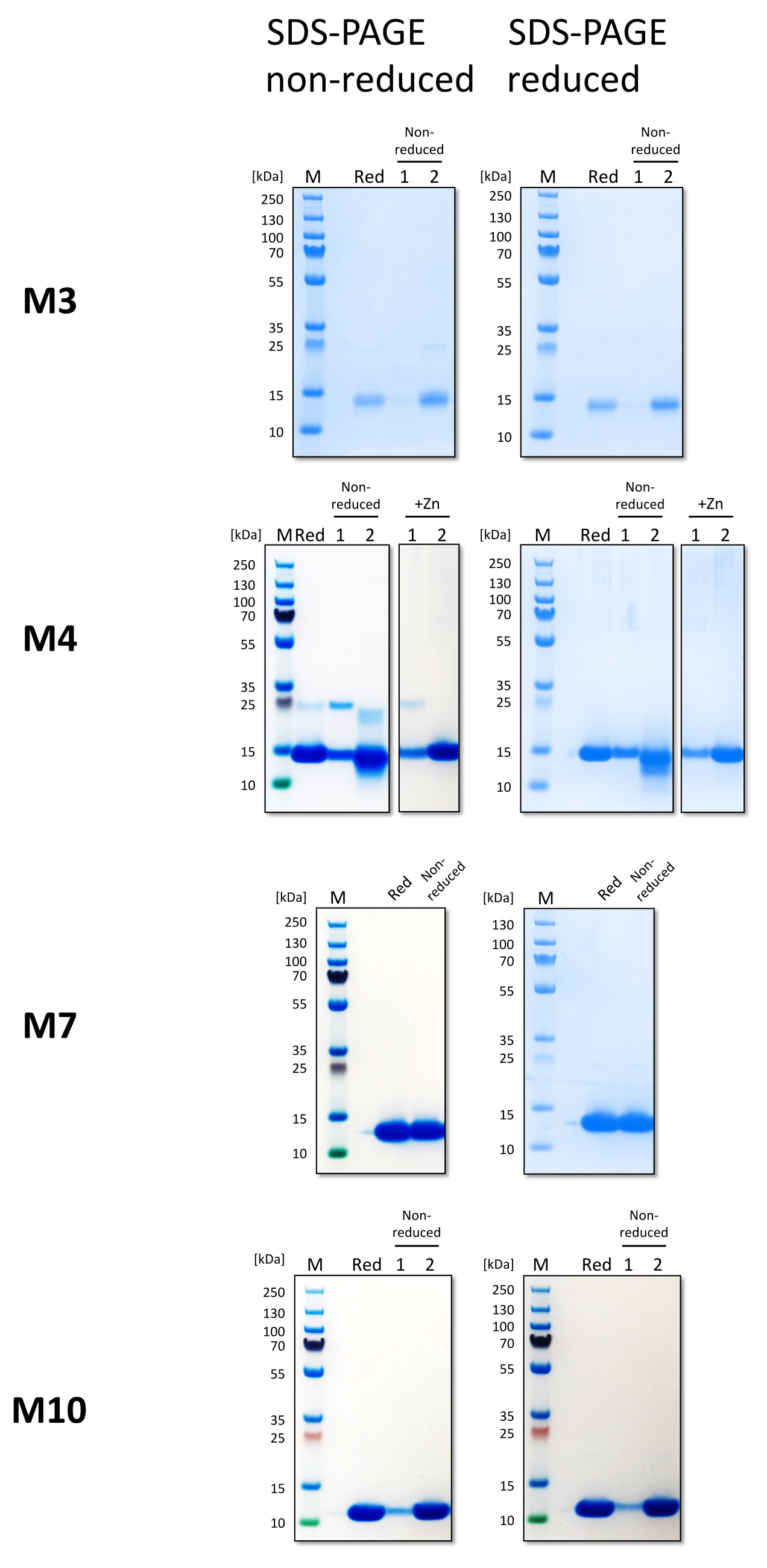

Supplement: S6 Fig — Row annotations 1 and 2 refer to dimeric peak (1) and monomeric peak (2). Data for M4 has already been shown in Fig 4. (TIF) [file pone.0226693.s006.tif]
