# Supplementary material for: Structural diversity in the atomic resolution 3D fingerprint of the titin M-band segment
Source: PLoS One. 2019 Dec 19;14(12):e0226693. doi: 10.1371/journal.pone.0226693 (PMC6922384; doi:10.1371/journal.pone.0226693)
Supplement: S1 Table — (DOCX) [file pone.0226693.s007.docx]

| **Domain** | **Residue range**  **(Q8WZ42)** | **Vector** | **Tags** | ***Escherichia coli* strain** |
| --- | --- | --- | --- | --- |
| **M1** | 32496-32590 | pET-M11 | N-6His-TEV | BL21 (DE3) CodonPlus-RIL |
| **M3** | 32712-32816 | pET-Z2-1a | N-6His-Z2-TEV | BL21 (DE3) CodonPlus-RIL |
| **M4** | 33293-33395 | pET-M14 | N-6His-3C | BL21 (DE3) |
| **M7** | 33773-33871 | pCDFM-14 | N-6His-3C | BL21 star (DE3) pRARE2 |
| **M10** | 34252–34350 | pET-M14 | N-6His-3C | BL21 star (DE3) pRARE2 |
