# Supplementary material for: Structural diversity in the atomic resolution 3D fingerprint of the titin M-band segment
Source: PLoS One. 2019 Dec 19;14(12):e0226693. doi: 10.1371/journal.pone.0226693 (PMC6922384; doi:10.1371/journal.pone.0226693)
Supplement: S2 Table — Homology models were calculated using SWISS-MODEL protein structure homology-modeling server [44]. Sequence similarity, Sequence coverage and Global Model Quality Estimate (GMQE) were calculated by the SWISS-MODEL server. (DOCX) [file pone.0226693.s008.docx]

| **M-domain (model)** | **M-domain (template)** | **PDB** | **Sequence  similarity** | **Sequence  coverage** | **Global Model Quality Estimation (GMQE)** |
| --- | --- | --- | --- | --- | --- |
| M2 | M7 | 3puc | 0.36 | 0.97 | 0.7 |
| M6 | M7 | 3puc | 0.34 | 0.94 | 0.66 |
| M8 | M4 | 3qp3 | 0.31 | 0.94 | 0.64 |
| M9 | M3 | 6hci | 0.37 | 0.99 | 0.75 |
