# Supplementary material for: Structural diversity in the atomic resolution 3D fingerprint of the titin M-band segment
Source: PLoS One. 2019 Dec 19;14(12):e0226693. doi: 10.1371/journal.pone.0226693 (PMC6922384; doi:10.1371/journal.pone.0226693)
Supplement: S3 Table — Where multiple protein chains are found, chain A was used. (DOCX) [file pone.0226693.s009.docx]

| **M-band domain** | **PDB ID** | **Q8WZ42 (**UniProt**, N)** | **N (PDB)** | **Q8WZ42 (**UniProt**, C)** | **C (PDB)** |
| --- | --- | --- | --- | --- | --- |
| M1 | 2bk8 | 32497 | 8 | 32590 | 101 |
| M3 | 6hci | 32718 | 3 | 32816 | 101 |
| M4 | 3qp3 | 33294 | 0 | 33393 | 99 |
| M4 | 6h4l |  |  |  |  |
| M5 | 1ncu | 33483 | 1 | 33579 | 97 |
| M7 | 3puc | 33774 | 2 | 33870 | 98 |
| M10 | 3q5o | 34252 | 4 | 34350 | 101 |
