# Supplementary material for: Structural diversity in the atomic resolution 3D fingerprint of the titin M-band segment
Source: PLoS One. 2019 Dec 19;14(12):e0226693. doi: 10.1371/journal.pone.0226693 (PMC6922384; doi:10.1371/journal.pone.0226693)
Supplement: S4 Table — Maximum accessible surface area of cysteine side chain: 46.6 Å2. Cysteine surface accessibility was calculated using pymol (The PyMOL Molecular Graphics System, Version 2.0 Schrödinger, LLC.). Where applicable, mean and S.D. were calculated with program Excel (Microsoft Corporation, Redmond, WA) from different domain copies available in the pdb file (M3: four copies; M4: three copies; M10: two copies). (DOCX) [file pone.0226693.s010.docx]

| **Domain** | **Residue** | **Accessible surface area** | |
| --- | --- | --- | --- |
|  |  | **[Å^2^]** | **[%]** |
| M1 | C27 | 0.0 | 0.0 |
|  | C79 | 0.0 | 0.0 |
| M3 | C18 | 3.7 ± 0.6 | 8.0 |
|  | C25 | 23.5 ± 6.8 | 50.6 |
|  | C91 | 8.7 ± 1.7 | 18.7 |
| M4 | C20 | 37.7 ± 4.9 | 81.0 |
|  | C69 | 0.0 ± 0.0 | 0.0 |
|  | C81 | 0.0 ± 0.0 | 0.0 |
| M7 | C79 | 0.0 | 0.0 |
|  | C92 | 0.0 | 0.0 |
| M10 | C28 | 0.8 ± 1.1 | 1.7 |
|  | C42 | 37.0 ± 1.3 | 79.5 |
