# Supplementary material for: Structural diversity in the atomic resolution 3D fingerprint of the titin M-band segment
Source: PLoS One. 2019 Dec 19;14(12):e0226693. doi: 10.1371/journal.pone.0226693 (PMC6922384; doi:10.1371/journal.pone.0226693)
Supplement: S5 Table — Mean and standard deviations of the free thiol titrations were calculated with program Excel (Microsoft Corporation, Redmond, WA) from three independent repeats of an experiment. (DOCX) [file pone.0226693.s011.docx]

| **M-band domain** | **Free thiol content** |
| --- | --- |
| M3 | 2.82±0.03 |
| M4 | 1.17±0.09 |
| M7 | 0.17±0.00 |
| M10 | 1.08±0.08 |
