# Supplementary material for: Structural diversity in the atomic resolution 3D fingerprint of the titin M-band segment
Source: PLoS One. 2019 Dec 19;14(12):e0226693. doi: 10.1371/journal.pone.0226693 (PMC6922384; doi:10.1371/journal.pone.0226693)
Supplement: S6 Validation — (PDF) [file pone.0226693.s029.pdf]

Full wwPDB X-ray Structure Validation Report (i)

Mar 8, 2018 – 05:28 pm GMT

PDB ID	:	3Q5O
Title	:	Crystal structure of human titin domain M10
Authors	:	Sauer, F.; Wilmanns, M.
Deposited on	:	2010-12-29
Resolution	:	2.05 Å(reported)

This is a Full wwPDB X-ray Structure Validation Report for a publicly released PDB entry.

We welcome your comments at *validation@mail.wwpdb.org*
A user guide is available at
https://www.wwpdb.org/validation/2017/XrayValidationReportHelp
with specific help available everywhere you see the (i) symbol.

The following versions of software and data (see references (i)) were used in the production of this report:

MolProbity	:	4.02b-467
Mogul	:	1.7.3 (157068), CSD as539be (2018)
Xtriage (Phenix)	:	1.13
EDS	:	trunk30967
Percentile statistics	:	20171227.v01 (using entries in the PDB archive December 27th 2017)
Refmac	:	5.8.0158
CCP4	:	7.0 (Gargrove)
Ideal geometry (proteins)	:	Engh & Huber (2001)
Ideal geometry (DNA, RNA)	:	Parkinson et al. (1996)
Validation Pipeline (wwPDB-VP)	:	trunk30967

1 Overall quality at a glance ⓘ

The following experimental techniques were used to determine the structure:
X-RAY DIFFRACTION

The reported resolution of this entry is 2.05 Å.

Percentile scores (ranging between 0-100) for global validation metrics of the entry are shown in the following graphic. The table shows the number of entries on which the scores are based.

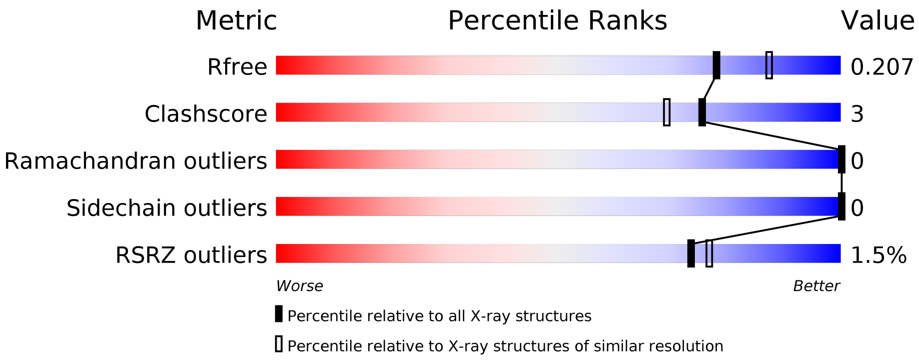

Metric	Whole archive (#Entries)	Similar resolution (#Entries, resolution range(Å))
R_{free}	111664	1449 (2.04-2.04)
Clashscore	122126	1524 (2.04-2.04)
Ramachandran outliers	120053	1512 (2.04-2.04)
Sidechain outliers	120020	1512 (2.04-2.04)
RSRZ outliers	108989	1429 (2.04-2.04)

The table below summarises the geometric issues observed across the polymeric chains and their fit to the electron density. The red, orange, yellow and green segments on the lower bar indicate the fraction of residues that contain outliers for >=3, 2, 1 and 0 types of geometric quality criteria. A grey segment represents the fraction of residues that are not modelled. The numeric value for each fraction is indicated below the corresponding segment, with a dot representing fractions <=5% The upper red bar (where present) indicates the fraction of residues that have poor fit to the electron density. The numeric value is given above the bar.

Mol	Chain	Length	Quality of chain
1	A	100	2% / 92% 8%
1	B	100	% / 93% 7%

2 Entry composition (i)

There are 3 unique types of molecules in this entry. The entry contains 1663 atoms, of which 0 are hydrogens and 0 are deuteriums.

In the tables below, the ZeroOcc column contains the number of atoms modelled with zero occupancy, the AltConf column contains the number of residues with at least one atom in alternate conformation and the Trace column contains the number of residues modelled with at most 2 atoms.

- Molecule 1 is a protein called Titin.

Mol	Chain	Residues	Atoms					ZeroOcc	AltConf	Trace
			Total	C	N	O	S			
1	A	100	748	466	126	153	3	0	1	0
1	B	100	743	463	126	151	3	0	0	0

There are 4 discrepancies between the modelled and reference sequences:

Chain	Residue	Modelled	Actual	Comment	Reference
A	1	GLY	-	EXPRESSION TAG	UNP Q8WZ42
A	2	PRO	-	EXPRESSION TAG	UNP Q8WZ42
B	1	GLY	-	EXPRESSION TAG	UNP Q8WZ42
B	2	PRO	-	EXPRESSION TAG	UNP Q8WZ42

- Molecule 2 is SULFATE ION (three-letter code: SO4) (formula: O_4S).

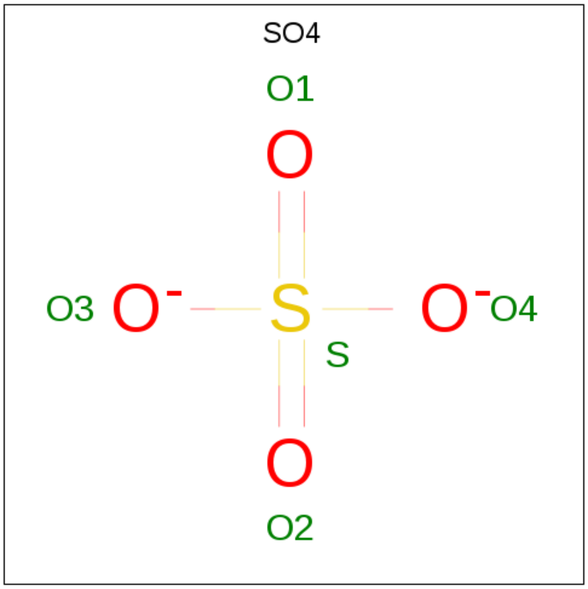

Mol	Chain	Residues	Atoms	ZeroOcc	AltConf
2	A	1	Total O S<br>5 4 1	0	0
2	B	1	Total O S<br>5 4 1	0	0
2	B	1	Total O S<br>5 4 1	0	0
2	B	1	Total O S<br>5 4 1	0	0

- Molecule 3 is water.

Mol	Chain	Residues	Atoms	ZeroOcc	AltConf
3	A	69	Total O<br>69 69	0	0
3	B	83	Total O<br>83 83	0	0

3 Residue-property plots (i)

These plots are drawn for all protein, RNA and DNA chains in the entry. The first graphic for a chain summarises the proportions of the various outlier classes displayed in the second graphic. The second graphic shows the sequence view annotated by issues in geometryand electron density. Residues are color-coded according to the number of geometric quality criteria for which they contain at least one outlier: green = 0, yellow = 1, orange = 2 and red = 3 or more. A red dot above a residue indicates a poor fit to the electron density (RSRZ > 2). Stretches of 2 or more consecutive residues without any outlier are shown as a green connector. Residues present in the sample, but not in the model, are shown in grey.

- Molecule 1: Titin

Chain A:
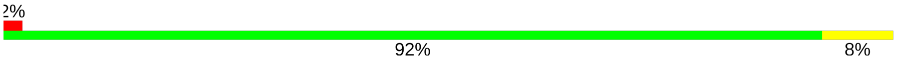

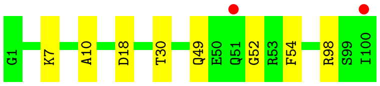

- Molecule 1: Titin

Chain B:
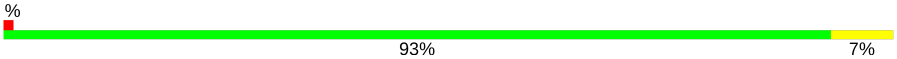

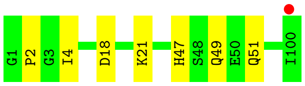

4 Data and refinement statistics (i)

Property	Value	Source
Space group	P 63 2 2	Depositor
Cell constants<br>a, b, c, α, β, γ	118.77Å 118.77Å 85.22Å<br>90.00° 90.00° 120.00°	Depositor
Resolution (Å)	48.72 – 2.05<br>48.72 – 2.05	Depositor<br>EDS
% Data completeness<br>(in resolution range)	99.2 (48.72-2.05)<br>99.3 (48.72-2.05)	Depositor<br>EDS
R_{merge}	0.05	Depositor
R_{sym}	(Not available)	Depositor
$< I/\sigma(I) >$ [1]	4.04 (at 2.05Å)	Xtriage
Refinement program	PHENIX (phenix.refine: 1.6.4_486)	Depositor
R, R_{free}	0.189 , 0.215<br>0.184 , 0.207	Depositor<br>DCC
R_{free} test set	1060 reflections (4.68%)	wwPDB-VP
Wilson B-factor (Å^2)	32.0	Xtriage
Anisotropy	0.718	Xtriage
Bulk solvent k_{sol}(e/Å^3), B_{sol}(Å^2)	0.32 , 48.2	EDS
L-test for twinning[2]	$< \|L\| > = 0.46$, $< L^2 > = 0.28$	Xtriage
Estimated twinning fraction	No twinning to report.	Xtriage
F_o,F_c correlation	0.96	EDS
Total number of atoms	1663	wwPDB-VP
Average B, all atoms (Å^2)	42.0	wwPDB-VP

Xtriage's analysis on translational NCS is as follows: *The largest off-origin peak in the Patterson function is 6.72% of the height of the origin peak. No significant pseudotranslation is detected.*

[1]Intensities estimated from amplitudes.

[2]Theoretical values of $< |L| >$, $< L^2 >$ for acentric reflections are 0.5, 0.333 respectively for untwinned datasets, and 0.375, 0.2 for perfectly twinned datasets.

5 Model quality (i)

5.1 Standard geometry (i)

Bond lengths and bond angles in the following residue types are not validated in this section: SO4

The Z score for a bond length (or angle) is the number of standard deviations the observed value is removed from the expected value. A bond length (or angle) with $|Z| > 5$ is considered an outlier worth inspection. RMSZ is the root-mean-square of all Z scores of the bond lengths (or angles).

Mol	Chain	Bond lengths		Bond angles	
		RMSZ	$\#|Z| >5$	RMSZ	$\#|Z| >5$
1	A	0.35	0/765	0.54	1/1039 (0.1%)
1	B	0.43	0/757	0.54	0/1028
All	All	0.39	0/1522	0.54	1/2067 (0.0%)

There are no bond length outliers.

All (1) bond angle outliers are listed below:

Mol	Chain	Res	Type	Atoms	Z	Observed(^{o})	Ideal(^{o})
1	A	10	ALA	CB-CA-C	5.15	117.82	110.10

There are no chirality outliers.

There are no planarity outliers.

5.2 Too-close contacts (i)

In the following table, the Non-H and H(model) columns list the number of non-hydrogen atoms and hydrogen atoms in the chain respectively. The H(added) column lists the number of hydrogen atoms added and optimized by MolProbity. The Clashes column lists the number of clashes within the asymmetric unit, whereas Symm-Clashes lists symmetry related clashes.

Mol	Chain	Non-H	H(model)	H(added)	Clashes	Symm-Clashes
1	A	748	0	725	6	0
1	B	743	0	721	4	0
2	A	5	0	0	0	0
2	B	15	0	0	1	0
3	A	69	0	0	0	1
3	B	83	0	0	0	0
All	All	1663	0	1446	10	1

The all-atom clashscore is defined as the number of clashes found per 1000 atoms (including hydrogen atoms). The all-atom clashscore for this structure is 3.

All (10) close contacts within the same asymmetric unit are listed below, sorted by their clash magnitude.

Atom-1	Atom-2	Interatomic distance (Å)	Clash overlap (Å)
1:A:49:GLN:HE21	1:A:52:GLY:HA3	1.65	0.60
1:A:49:GLN:HG3	1:A:52:GLY:HA3	1.87	0.56
1:A:7:LYS:HB3	1:A:30:THR:CG2	2.35	0.56
1:A:18:ASP:OD1	1:A:98:ARG:HD3	2.06	0.55
1:B:2:PRO:O	1:B:4:ILE:HG13	2.08	0.53
1:B:18:ASP:HB2	1:B:21:LYS:HD2	1.93	0.50
1:A:49:GLN:HG3	1:A:52:GLY:CA	2.46	0.46
1:A:49:GLN:HG2	1:A:54:PHE:O	2.16	0.45
1:B:49:GLN:HG3	1:B:51:GLN:O	2.17	0.44
1:B:47:HIS:HE1	2:B:101:SO4:O4	2.03	0.41

All (1) symmetry-related close contacts are listed below. The label for Atom-2 includes the symmetry operator and encoded unit-cell translations to be applied.

Atom-1	Atom-2	Interatomic distance (Å)	Clash overlap (Å)
3:A:114:HOH:O	3:A:114:HOH:O[9_556]	2.15	0.05

5.3 Torsion angles (i)

5.3.1 Protein backbone (i)

In the following table, the Percentiles column shows the percent Ramachandran outliers of the chain as a percentile score with respect to all X-ray entries followed by that with respect to entries of similar resolution.

The Analysed column shows the number of residues for which the backbone conformation was analysed, and the total number of residues.

Mol	Chain	Analysed	Favoured	Allowed	Outliers	Percentiles	
1	A	99/100 (99%)	98 (99%)	1 (1%)	0	100	100
1	B	98/100 (98%)	97 (99%)	1 (1%)	0	100	100
All	All	197/200 (98%)	195 (99%)	2 (1%)	0	100	100

There are no Ramachandran outliers to report.

5.3.2 Protein sidechains (i)

In the following table, the Percentiles column shows the percent sidechain outliers of the chain as a percentile score with respect to all X-ray entries followed by that with respect to entries of similar resolution.

The Analysed column shows the number of residues for which the sidechain conformation was analysed, and the total number of residues.

Mol	Chain	Analysed	Rotameric	Outliers	Percentiles	
1	A	83/85 (98%)	83 (100%)	0	100	100
1	B	82/85 (96%)	82 (100%)	0	100	100
All	All	165/170 (97%)	165 (100%)	0	100	100

There are no protein residues with a non-rotameric sidechain to report.

Some sidechains can be flipped to improve hydrogen bonding and reduce clashes. All (5) such sidechains are listed below:

Mol	Chain	Res	Type
1	A	47	HIS
1	A	49	GLN
1	A	58	ASN
1	B	47	HIS
1	B	49	GLN

5.3.3 RNA (i)

There are no RNA molecules in this entry.

5.4 Non-standard residues in protein, DNA, RNA chains (i)

There are no non-standard protein/DNA/RNA residues in this entry.

5.5 Carbohydrates (i)

There are no carbohydrates in this entry.

5.6 Ligand geometry (i)

4 ligands are modelled in this entry.

In the following table, the Counts columns list the number of bonds (or angles) for which Mogul statistics could be retrieved, the number of bonds (or angles) that are observed in the model and the number of bonds (or angles) that are defined in the Chemical Component Dictionary. The Link column lists molecule types, if any, to which the group is linked. The Z score for a bond length (or angle) is the number of standard deviations the observed value is removed from the expected value. A bond length (or angle) with $|Z| > 2$ is considered an outlier worth inspection. RMSZ is the root-mean-square of all Z scores of the bond lengths (or angles).

Mol	Type	Chain	Res	Link	Bond lengths			Bond angles		
					Counts	RMSZ	$\#\|Z\| > 2$	Counts	RMSZ	$\#\|Z\| > 2$
2	SO4	A	102	-	4,4,4	0.16	0	6,6,6	0.08	0
2	SO4	B	101	-	4,4,4	0.18	0	6,6,6	0.11	0
2	SO4	B	102	-	4,4,4	0.16	0	6,6,6	0.10	0
2	SO4	B	103	-	4,4,4	0.17	0	6,6,6	0.06	0

In the following table, the Chirals column lists the number of chiral outliers, the number of chiral centers analysed, the number of these observed in the model and the number defined in the Chemical Component Dictionary. Similar counts are reported in the Torsion and Rings columns. '-' means no outliers of that kind were identified.

Mol	Type	Chain	Res	Link	Chirals	Torsions	Rings
2	SO4	A	102	-	-	0/0/0/0	0/0/0/0
2	SO4	B	101	-	-	0/0/0/0	0/0/0/0
2	SO4	B	102	-	-	0/0/0/0	0/0/0/0
2	SO4	B	103	-	-	0/0/0/0	0/0/0/0

There are no bond length outliers.

There are no bond angle outliers.

There are no chirality outliers.

There are no torsion outliers.

There are no ring outliers.

1 monomer is involved in 1 short contact:

Mol	Chain	Res	Type	Clashes	Symm-Clashes
2	B	101	SO4	1	0

5.7 Other polymers (i)

There are no such residues in this entry.

5.8 Polymer linkage issues (i)

There are no chain breaks in this entry.

6 Fit of model and data (i)

6.1 Protein, DNA and RNA chains (i)

In the following table, the column labelled '#RSRZ> 2' contains the number (and percentage) of RSRZ outliers, followed by percent RSRZ outliers for the chain as percentile scores relative to all X-ray entries and entries of similar resolution. The OWAB column contains the minimum, median, 95th percentile and maximum values of the occupancy-weighted average B-factor per residue. The column labelled 'Q< 0.9' lists the number of (and percentage) of residues with an average occupancy less than 0.9.

Mol	Chain	Analysed	<RSRZ>	#RSRZ>2			OWAB(Å^2)	Q<0.9
1	A	100/100 (100%)	-0.37	2 (2%)	65	69	24, 36, 71, 96	0
1	B	100/100 (100%)	-0.45	1 (1%)	82	84	22, 35, 71, 98	0
All	All	200/200 (100%)	-0.41	3 (1%)	73	77	22, 36, 71, 98	0

All (3) RSRZ outliers are listed below:

Mol	Chain	Res	Type	RSRZ
1	A	100	ILE	3.7
1	B	100	ILE	3.2
1	A	51	GLN	2.5

6.2 Non-standard residues in protein, DNA, RNA chains (i)

There are no non-standard protein/DNA/RNA residues in this entry.

6.3 Carbohydrates (i)

There are no carbohydrates in this entry.

6.4 Ligands (i)

In the following table, the Atoms column lists the number of modelled atoms in the group and the number defined in the chemical component dictionary. The B-factors column lists the minimum, median, 95th percentile and maximum values of B factors of atoms in the group. The column labelled 'Q< 0.9' lists the number of atoms with occupancy less than 0.9.

Mol	Type	Chain	Res	Atoms	RSCC	RSR	B-factors(Å^2)	Q<0.9
2	SO4	B	103	5/5	0.88	0.28	110,110,111,112	5
2	SO4	B	102	5/5	0.90	0.14	100,101,102,103	0

Continued on next page...

Continued from previous page...

Mol	Type	Chain	Res	Atoms	RSCC	RSR	B-factors(Å^2)	Q<0.9
2	SO4	A	102	5/5	0.92	0.13	106,107,108,108	0
2	SO4	B	101	5/5	0.96	0.25	78,81,84,86	0

6.5 Other polymers ⓘ

There are no such residues in this entry.
